# Dietary tryptophan deficiency and its supplementation compromises inflammatory mechanisms and disease resistance in a teleost fish

**DOI:** 10.1038/s41598-019-44205-3

**Published:** 2019-05-22

**Authors:** M. Machado, R. Azeredo, A. Domingues, S. Fernandez-Boo, J. Dias, L. E. C. Conceição, B. Costas

**Affiliations:** 1 Centro Interdisciplinar de Investigação Marinha e Ambiental (CIIMAR), Terminal de Cruzeiros do Porto de Leixões, Av. General Norton de Matos s/n, 4450-208 Matosinhos, Portugal; 2 Instituto de Investigação e Inovação em Saúde (i3S), Universidade do Porto, Rua Alfredo Allen, 208, 4200-135 Porto, Portugal; 3 Instituto de Ciências Biomédicas Abel Salazar (ICBAS-UP), Universidade do Porto, Rua de Jorge Viterbo Ferreira no. 228, 4050-313 Porto, Portugal; 4 Instituto de Biologia Molecular e Celular, Universidade do Porto, Rua Alfredo Allen, 208, 4200-135 Porto, Portugal; 5 Sparos Lda, Area Empresarial de Marim, Lote C, Olhão, Portugal

**Keywords:** Biological sciences, Immunology

## Abstract

Tryptophan participates on several physiological mechanisms of the neuroendocrine-immune network and plays a critical role in macrophages and lymphocytes function. This study intended to evaluate the modulatory effects of dietary tryptophan on the European seabass (*Dicentrarchus labrax*) immune status, inflammatory response and disease resistance to *Photobacterium damselae piscicida*. A tryptophan deficient diet (NTRP); a control diet (CTRL); and two other diets supplemented with tryptophan at 0.13% (TRP13) and 0.17% (TRP17) of feed weight were formulated. Fish were sampled at 2 and 4 weeks of feeding and the remaining were i.p. injected with *Phdp* (3 × 10^6^ cfu/fish) at 4 weeks and the inflammatory response (at 4, 24, 48 and 72 hours post-infection) as well as survival were evaluated. Results suggest that fish immune status was not altered in a tryptophan deficient scenario whereas in response to an inflammatory insult, plasma cortisol levels increased and the immune cell response was compromised, which translated in a lower disease resistance. When dietary tryptophan was offered 30% above its requirement level, plasma cortisol increased and, in response to bacterial infection, a decrease in lymphocytes, monocytes/macrophages and several immune-related genes was observed, also compromising at some degree fish disease resistance.

## Introduction

Teleost requirements for amino acids (AA) are generally settled by means of optimal growth. However, the assembling of several physiological challenges may require extra AA for metabolic processes, thus demand of certain nutrients may increase. In fact, AA requirements may increase as a result of metabolic deviations associated with stress response and infection^1^. Nutrients are known to influence several aspects of the immune system^2^, and it is likely that immune mechanisms may be modulated through nutritional strategies^3^. As in mammals, tryptophan is an essential AA with important roles for protein synthesis and as precursor of several compounds with a wide range of effects in the modulation of stress response, antioxidant system, behavioural response and immune system^4,5^. Most tryptophan catabolism occurs through the kynurenine pathway in the liver and is mediated by tryptophan 2,3-dioxygenase (TDO). However, the production of niacin, for the synthesis of NAD^+^, from tryptophan catabolism appears to be limited in fish^6^. Thus, the kynurenine – niacin pathway in the liver seems to be mainly directed to the control of tryptophan levels^4,6^. The same pathway of tryptophan catabolism is present in macrophages and is mediated by indoleamine 2,3-dioxygenase (IDO), which catalyses the first and rate limiting step of the tryptophan catabolism along the kynurenine pathway. IDO relies on the availability of tryptophan^7^ and its induction by inflammatory stimuli as interferon-γ and cytokines^8^. In IDO^+^ cells, tryptophan contributes to metabolic immune regulation in three different ways: (1) mediating anti-microbial effects by tryptophan depletion from the extracellular environment, reducing its availability to microorganisms^9^; (2) its metabolites as 3-hydroxykynurenine, 3-hydroxyanthranilic acid and quinolinic acid are able to regulate T-cell function; and (3) the same metabolites set up a protector system acting locally to the removal of superoxide radicals modulating oxidative status^10^, thus creating conditions that favour immune suppression and tolerance.

The interaction of neuroendocrine and immune machineries is a recognized phenomenon that allow animals to better cope with the disturbance of homeostasis as both share physiological pathways. Both immune and endocrine cells share common receptors while different hormones and cytokines are involved in the same mechanisms^11,12^. Glucocorticoids are a clear example of this interaction, as they modulate the secretion of cytokines and has become evident that most immune cell types can be affected by them^13^. In particular, cortisol can have a clear effect on multiple characteristics of the immune defence mechanism in fish, as reviewed by Hoseini, *et al*.^4^. For instance, cortisol was able to inhibit the expression of pro-inflammatory cytokines and nitric oxide upon lipopolysaccharides induction^14,15^ or induction of apoptosis and inhibition of immune cells proliferation^16^ in fish. Therefore, an endocrine-immune perspective should be considered when evaluating tryptophan immune-nutrition, as it presents roles in both systems. Serotonin (5-HT) is produced from tryptophan in the central nervous system and in the gastrointestinal tract and its synthesis controls the adrenocorticotropic hormone release, regulating cortisol production^17,18^. Together with 5-HT, the tryptophan metabolites melatonin and N-acetylserotonin, appear to enhance host immunity by reducing the production of superoxide, scavenging free radicals and attenuation the production of pro-inflammatory cytokines^19^.

The ideal inflammatory response is rapid, specific and self-limiting^20^. In this context, and knowing that tryptophan’s role during infection is mainly related to regulatory processes leading to anti-inflammatory signalling molecules, this AA presents itself as a tentative immunomodulator during the development of inflammation and its resolution. However, the potential benefit of tryptophan supplementation for animal health management is not fully demonstrated, and its immunomodulatory role in fish must be further investigated. Tryptophan immune-nutrition also adds a practical point to modern animal production as a strategy to counteract the deleterious effect of an aggravated and uncontrolled immune response. Therefore, the present study aimed to evaluate the effects of dietary tryptophan deficiency and supplementation on the European seabass immune status, inflammatory response and disease resistance.

## Results

### Immune status

#### Fish growth performance

Thirty six fish per group (12 fish/replicate) were sampled and weighted after feeding with the experimental diets for 4 weeks in order to evaluate the effect of the diets on the growth performance. Experimental diets were well accepted and there were no mortalities throughout the feeding trial. Within each group, no differences in final weight were found between replicate and experimental diets in any of the growth parameters evaluated (Table 1). Taken together, these results suggest that tryptophan deficiency or supplementation to a diet that ensures the established nutritional requirements to the target species, does not affect growth performance.

**Table 1:** Data on the initial and final weight and growth performance of European seabass sampled at 4 weeks after being fed four different diets.

Parameters		Dietary treatments			
		NTRP	CTRL	TRP 13	TRP 17
Initial weight	g	9.68 ± 0.20	9.76 ± 0.39	10.16 ± 1.28	9.11 ± 0.08
Final weight	g	11.94 ± 2.00	11.75 ± 1.54	11.88 ± 1.73	11.86 ± 1.74

#### Haematology and peripheral leucocyte responses

Blood of 9 fish from each group (3 per replicate) was sampled at 2 and 4 weeks and was used for evaluation of hematological parameters. Both haematological and peripheral leucocyte responses were not significantly altered by dietary treatments. Despite the lack of differences found in RBC, haemoglobin concentration and MCH decreased with time regardless dietary treatments while the opposite pattern was observed for total WBC, thrombocytes and lymphocytes concentration (Tables 2 and 3).

**Table 2:** Haemoglobin, mean corpuscular haemoglobin (MCH), red blood cells (RBC) and white blood cells (WBC) in European seabass fed dietary treatments during 2 and 4 weeks.

Parameters		Dietary treatments							
		NTRP		CTRL		TRP 13		TRP 17	
		2 weeks	4 weeks	2 weeks	4 weeks	2 weeks	4 weeks	2 weeks	4 weeks
Haemoglobin	(g dl)	1.07 ± 0.15	0.87 ± 0.33	1.13 ± 0.27	0.70 ± 0.25	1.17 ± 0.10	0.72 ± 0.27	1.01 ± 0.15	0.80 ± 0.10
MCH	(pg cell^−1^)	5.55 ± 1.10	4.30 ± 1.40	5.67 ± 1.18	3.48 ± 0.66	5.66 ± 0.64	3.39 ± 1.00	5.43 ± 0.94	3.62 ± 0.62
RBC	(×10^6^ µl^−1^)	1.98 ± 0.32	2.03 ± 0.37	2.03 ± 0.48	1.96 ± 0.48	2.09 ± 0.29	2.14 ± 0.45	1.87 ± 0.22	2.23 ± 0.24
WBC	(×10^4^ µl^−1^)	7.10 ± 2.12	9.14 ± 3.57	6.72 ± 1.26	9.36 ± 2.32	7.51 ± 1.84	8.33 ± 2.62	7.56 ± 1.42	10.10 ± 1.82
**Two-way ANOVA**									
**Parameters**		**Time**	**Diet**	**Time** × **Diet**					
Haemoglobin		<0.001	ns	ns					
MCH		<0.001	ns	ns					
RBC		ns	ns	ns					
WBC		<0.001	ns	ns					

Values are presented as means ± SD (n = 9). P-values from two-way ANOVA (p ≤ 0.05).

**Table 3:** Absolute values of peripheral blood leucocytes (thrombocytes, lymphocytes, monocytes and neutrophils) of European seabass fed dietary treatments during 2 and 4 weeks.

Parameters		Dietary treatments							
		NTRP		CTRL		TRP 13		TRP 17	
		2 weeks	4 weeks	2 weeks	4 weeks	2 weeks	4 weeks	2 weeks	4 weeks
Thrombocytes	(×10^4^ µl^−1^)	4.56 ± 1.44	4.87 ± 1.53	3.95 ± 0.66	5.62 ± 1.38	4.49 ± 0.86	4.85 ± 1.30	4.11 ± 0.81	6.07 ± 0.84
Lymphocytes		2.25 ± 0.81	3.97 ± 2.18	2.49 ± 0.83	3.30 ± 1.22	2.70 ± 0.89	3.18 ± 1.48	3.08 ± 0.89	3.82 ± 1.21
Monocytes		0.16 ± 0.08	0.16 ± 0,07	0.21 ± 0.18	0.17 ± 0.15	0.23 ± 0.28	0.22 ± 0.14	0.19 ± 0.17	0.15 ± 0.11
Neutrophils		0.14 ± 0.09	0.14 ± 0.08	0.10 ± 0.05	0.18 ± 0.12	0.10 ± 0.11	0.08 ± 0.04	0.18 ± 0.15	0.10 ± 0.06
**Two-way ANOVA**									
**Parameters**		**Time**	**Diet**	**Time** × **Diet**					
Thrombocytes		<0.001	ns	ns					
Lymphocytes		0.003	ns	ns					
Monocytes		ns	ns	ns					
Neutrophils		ns	ns	ns					

Values are presented as means ± SD (n = 9). P-values from two-way ANOVA (p ≤ 0.05).

#### Humoral innate immune response

Plasma innate humoral response was evaluated using 36 fish, collected from each experimental group (12 per replicate) and, for reasons of quantity limitation, the plasma from each 3 fish was pooled. Humoral innate immune parameters assessed in plasma are presented in Table 7.

Few differences were found regarding humoral immune parameters and cortisol in plasma. While peroxidase concentration was found to increase with time, dietary treatments did not change lysozyme, peroxidase, alternative complement pathway nor bactericidal activities. In contrast, plasma cortisol levels were higher in fish fed TRP 30 relative to fish fed CTRL and NTRP after 4 weeks of feeding (Table 4).

**Table 4:** Plasma lysozyme, peroxidase, alternative complement pathway (expressed as ACH50) and bactericidal activities as well as cortisol levels of European seabass fed dietary treatments during 2 and 4 weeks.

Parameters		Dietary treatments							
		NTRP		CTRL		TRP 13		TRP 17	
		2 weeks	4 weeks	2 weeks	4 weeks	2 weeks	4 weeks	2 weeks	4 weeks
Lysozyme	(µg ml^−1^)	8.09 ± 1.30	8.11 ± 3,90	9.58 ± 1.87	7.86 ± 2.23	9.07 ± 2.64	8.39 ± 1.94	7.42 ± 1.16	10.97 ± 3.36
Peroxidase	(units ml^−1^)	95.55 ± 9.21	118.63 ± 19.73	101.04 ± 113.19	123.71 ± 13.94	97.37 ± 16.96	135.49 ± 27.82	90.84 ± 18.05	139.23 ± 29.92
ACH50	(units ml^−1^)	66.09 ± 14.55	92.16 ± 26.76	115.92 ± 60.05	66.74 ± 10.31	86.02 ± 21.51	86.84 ± 22.67	103.3 ± 35.62	119.60 ± 30.88
Bactericidal activity	(%)	12.64 ± 8.42	26.38 ± 11.86	24.24 ± 13,29	28.32 ± 7.64	17.23 ± 7.92	23.76 ± 14.90	16.24 ± 7.48	17.43 ± 11.36
Cortisol	(ng ml^−1^)	26.43 ± 10.12	25.99 ± 4.40^b^	29.71 ± 16.07	27.74 ± 3.09^b^	24.57 ± 13.49	37.00 ± 6.67^a,b^	27.57 ± 8.73	43.60 ± 12.88^a^
**Two-way ANOVA**									
**Parameters**		**Time**	**Diet**	**Time** × **Diet**					
Lysozyme		ns	ns	ns					
Peroxidase		<0.001	ns	ns					
ACH50		ns	ns	ns					
Bactericidal activity		ns	ns	ns					
Cortisol		0.017	ns	0.044					

Values are presented as means ± SD (n = 12). P-values from two-way ANOVA (p ≤ 0.05). If interaction was significant, Tukey post hoc test was used to identify differences in the experimental treatments. Different lowercase letters stand for significant differences among dietary treatments for the same time.

#### Head-kidney gene expression

The evaluation of genes related to immune response as well as the tryptophan metabolism role in immune response (Table 5) was performed in the head-kidney. The cDNA was isolated from collected from 9 fish from each group (3 per replicate).

**Table 5:** Immune-related genes analysed by real-time PCR.

Gene	Acronym	Gene	Acronym
Elongation-factor 1β (House-Keeping)	*ef1α*	Cluster of differentiation 8 beta	*cd8β*
Interleukin 1 β	*il1β*	Heat shock protein 70	*hsp70*
Interleukin 10	*il10*	Heat shock protein 90	*hsp90*
Interleukin 8	*il8*	Matrix-metalloproteinase 9	*mmp9*
Transforming growth factor-beta	*tgfβ*	Dicentracin	*dicent*
Superoxide dismutase	*sod*	Glucocorticoid receptor 1	*gr1*
Ciclo-oxigenase 2	*cox 2*	Macrophage migration inhibitory factor	*mif*
Melanocortin 2 receptor	*mc2r*	Caspase 3	*casp3*
Interferon gamma	*ifn-γ*	Indoleamine 2	*ido2*
Cluster of differentiation 3 zeta chain	*c3zeta*	Arylformamidase-like	*afmid*
Macrophage colony-stimulating factor 1 receptor 1	*mcsf1r1*		

All data regarding gene expression during the feeding trial is presented in Table S1 as Supplementary Data. The normalized expression levels of *il1β*, *il10*, *sod*, *cox*2, *m*2*cr*, *ifn-γ*, *c3zeta*, *hsp70*, *hsp90*, *mmp9*, *gr1*, *mif*, *ido* and *afmid* showed a decrease between both sampling times. *dicent* mRNA expression level decreased with time only in those fish fed CTRL, while fish fed NTRP and CTRL presented increased *dicent* transcripts compared to fish fed TRP 13 and TRP 17 at 2 weeks.

### Bacterial challenge

**Figure 1:**
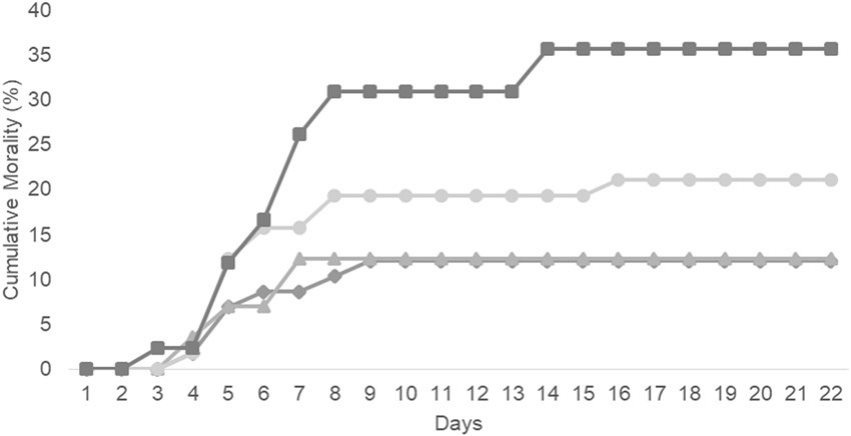
Cumulative mortality (%) of European seabass fed NTRP (), CTRL (), TRP 13 () and TRP 17 () for 4 weeks and subsequently infected with Phdp (n = 60).

To evaluate the effect of a diet deficient and supplemented with tryptophan during a bacterial infection, 60 fish from each group (20/replicate) were inoculated with *Phdp* and their mortality followed for 3 weeks (Fig. 1).

Significant differences were found only found between NTRP and CTRL (X^2^ = 0.004), presenting the tryptophan deficient diet the highest cumulative mortality (35.71%) followed by fish fed TRP 17 (21.05%). Fish fed both CTRL and TRP13 presented a similar and lowest cumulative mortality (12.07 and 12.28%, accordingly).

### Infection response

To examine the effect that tryptophan deficiency and supplementation may have on the inflammatory response following *Phdp* infection, samples of blood, head kidney, and peritoneal exudates were collected at 4, 24, 48 and 72 hours post-infection from fish of each experimental group (6 fish from each experimental diet by time-point). Sampling point 4 weeks was used as time 0 h during time-course data analysis, as they represent unstimulated animal prior infection. Accordingly, the collected samples were used to analyze whether the diets deficient or supplemented with tryptophan, compared to the control diet, triggered hematological alterations and changes in the innate immune response and the expression of genes in the head kidney. Also, changes in the intraperitoneal leukocyte populations were observed.

#### Haematology and peripheral leucocyte responses

Haemoglobin concentration and MCH increased in a time dependent manner after infection regardless dietary treatments whereas RBC numbers were not affected (Table 6). In contrast, WBC concentration decreased immediately after infection (0 h relative to 4 h), and those numbers recovered at 24 h with a slightly increase at 48 h (Table 6).

**Table 6:** Haemoglobin, mean corpuscular haemoglobin (MCH), red blood cells (RBC) and white blood cells (WBC) in European seabass fed dietary treatments prior infection (0 h) and at 4, 24, 48 and 72 h after peritoneal injection with *Phdp*.

Parameters		Dietary treatments									
		NTRP					CTRL				
		0 h	4 h	24 h	48 h	72 h	0 h	4 h	24 h	48 h	72 h
Haemoglobin	(g dl)	0.87 ± 0.33	0.96 ± 0.26	1.23 ± 0.20	1.86 ± 0.37	1.68 ± 0.17	0.70 ± 0.25	0.77 ± 0.13	1.33 ± 0.32	2.12 ± 0.34	1.85 ± 0.31
MCH	(pg cell^−1^)	4.30 ± 1.40	6.24 ± 0.13	6.08 ± 1.32	9.82 ± 0.88	7.53 ± 3.52	3.48 ± 0.66	3.21 ± 0.83	6.95 ± 2.52	10.31 ± 2.16	18.52 ± 2.00
RBC	(×10^6^ µl^−1^)	2.03 ± 0.37	1.86 ± 0.74	2.06 ± 0.23	1.89 ± 0.32	1.81 ± 0.34	1.96 ± 0.48	2.45 ± 0.27	1.99 ± 0.23	2.11 ± 0.46	2.24 ± 0.40
WBC	(×10^4^ µl^−1^)	9.14 ± 3.57	5.12 ± 1.89	7.43 ± 1.45	9.38 ± 2.45	8.68 ± 2.49	9.36 ± 2.32	6.00 ± 1.44	6.92 ± 2.07	11.70 ± 3.30	11.47 ± 4.00
**Parameters**		**Dietary treatments**									
		**TRP 13**					**TRP 17**				
		**0** **h**	**4** **h**	**24** **h**	**48** **h**	**72** **h**	**0** **h**	**4** **h**	**24** **h**	**48** **h**	**72** **h**
Haemoglobin	(g dl)	0.72 ± 0.27	0.95 ± 0.23	1.12 ± 0.15	2.05 ± 0.40	1.94 ± 0.57	0.80 ± 0.10	0.80 ± 0.18	1.14 ± 0.27	2.15 ± 0.65	1.90 ± 0.57
MCH	(pg cell^−1^)	3.39 ± 1.00	4.10 ± 0.73	5.45 ± 0.63	10.45 ± 2.56	10.42 ± 2.64	3.62 ± 0.62	3.65 ± 0.72	5.76 ± 1.33	10.88 ± 3.90	10.49 ± 3.30
RBC	(×10^6^ µl^−1^)	2.14 ± 0.45	2.36 ± 0.58	2.08 ± 0.29	2.03 ± 0.45	1.87 ± 0.26	2.23 ± 0.24	2.20 ± 0.33	2.00 ± 0.31	2.07 ± 0.39	1.84 ± 0.22
WBC	(×10^4^ µl^−1^)	8.33 ± 2.62	6.05 ± 1.64	6.85 ± 1.30	11.43 ± 7.65	8.85 ± 2.25	10.10 ± 1.82	5.42 ± 1.44	7.10 ± 1.69	9.53 ± 3.51	8.92 ± 1.22
**Two-way ANOVA**											
**Parameters**		**Time**	**Diet**	**Time** × **Diet**	**Time**						
					**0** **h**	**4** **h**	**24** **h**	**48** **h**	**72** **h**		
Haemoglobin		<0.001	ns	ns	C	C	B	A	A		
MCH		<0.001	ns	ns	C	BC	B	A	A		
RBC		ns	ns	ns	—	—	—	—	—		
WBC		<0.001	ns	ns	AB	C	B	A	AB		

Values are presented as means ± SD (n = 9 for 0 h and n = 6 for the remaining times). P-values from two-way ANOVA (p ≤ 0.05). If interaction was significant, Tukey post hoc test was used to identify differences in the experimental treatments. Different capital letters indicate differences in time regardless diets.

Absolute values of peripheral blood leucocytes are presented in Table 7. A time effect was also observed in the differential leucocyte counting with a significantly decrease of thrombocytes and lymphocytes concentration after infection whereas these leucocytes recovered up to the initial values at 48 h. Peripheral lymphocytes numbers was significantly lower in fish fed TRP13 and TRP17 relative to fish fed CTRL regardless time. Monocytes concentration decreased in fish fed NTRP, TRP 13 and TRP 17 at 24 h compared to fish fed CTRL. Fish fed NTRP presented an enhanced concentration of monocytes at 72 h relative to 0 h, 4 h and 24 h, whereas at 24 and 72 h fish fed CTRL presented increased monocytes levels relative to 0 and 4 h. Moreover, fish fed TRP 13 and TRP 17 displayed higher monocytes concentration at 48 h and 72 h relative to the remaining sampling points. Circulating neutrophils numbers were higher in fish fed TRP 17 than in those fed NTRP at 24 h. Fish fed CTRL presented superior levels of neutrophils at 48 h relative to 0 and 4 h, while fish fed TRP 13 presented higher concentration at 24 and 48 h relative to 0 h and at 24 h relative to 72 h. An increased concentration of neutrophils was also observed in fish fed TRP 17 at 24 h relative to 0, 4 and 72 h and at 48 h relative to 0 h.

**Table 7:** Absolute values of peripheral blood leucocytes (thrombocytes, lymphocytes, monocytes and neutrophils) of European seabass fed dietary treatments prior infection (0 h) and at 4, 24, 48 and 72 h after peritoneal injection with *Phdp*.

Parameters		Dietary treatments											
		NTRP					CTRL						
		0 h	4 h	24 h	48 h	72 h	0 h	4 h	24 h	48 h	72 h		
Thrombocytes	(×10^4^ µl^−1^)	4.87 ± 1.53	3.60 ± 1.34	5.17 ± 1.02	5.35 ± 1.09	4.86 ± 1.29	5.62 ± 1.38	3.97 ± 0.85	4.89 ± 1.67	5.87 ± 1.30	6.34 ± 2.20		
Lymphocytes		3.97 ± 2.18	1.22 ± 0.43	1.65 ± 0.25	3.13 ± 1.54	2.78 ± 1.25	3.3 ± 1.22	1.91 ± 0.79	1.99 ± 0.80	4.45 ± 1.92	3.83 ± 1.70		
Monocytes		0.16 ± 0.07^£^	0.16 ± 0.13^£^	0.11 ± 0.09^b,£^	0.29 ± 0.09*^,£^	0.69 ± 0.31*	0.17 ± 0.15^£^	0.10 ± 0.09^£^	0.69 ± 0.27^a,^*	0.30 ± 0.15*^,£^	0.78 ± 0.46*		
Neutrophils		0.14 ± 0.08	0.24 ± 0.25	0.51 ± 0.30^b^	0.61 ± 0.17	0.36 ± 0.30	0.18 ± 0.12^£^	0.19 ± 0.07^£^	0.74 ± 0.27^a,b,^*^,£^	1.07 ± 0.44*	0.52 ± 0.17*^,£^		
**Parameters**		**Dietary treatments**											
		**TRP 13**					**TRP 17**						
		**0** **h**	**4** **h**	**24** **h**	**48** **h**	**72** **h**	**0** **h**	**4** **h**	**24** **h**	**48** **h**	**72** **h**		
Thrombocytes	(×10^4^ µl^−1^)	4.85 ± 1.30	4.10 ± 1.05	4.69 ± 0.91	6.03 ± 3.26	5.37 ± 1.27	6.07 ± 0.84	3.73 ± 1.12	2.91 ± 2.14	4.63 ± 1.30	5.59 ± 1.35		
Lymphocytes		3.18 ± 1.48	1.27 ± 0.61	1.19 ± 0.30	2.82 ± 1.41	1.88 ± 0.56	3.82 ± 1.21	1.15 ± 0.28	0.55 ± 0.50	2.72 ± 1.68	2.14 ± 0.73		
Monocytes		0.22 ± 0.14^£^	0.10 ± 0.07^£^	0.08 ± 0.07^b,£^	0.34 ± 0.21*	0.55 ± 0.29*	0.15 ± 0.11^£^	0.11 ± 0.05^£^	0.06 ± 0.02^b,£^	0.30 ± 0.18*	0.37 ± 0.28*		
Neutrophils		0.08 ± 0.04^§^	0.39 ± 0.10*^,£,§^	0.88 ± 0.31^a,b,*^	0.74 ± 0.51*^,£^	0.21 ± 0.07^£,§^	0.10 ± 0.06^§^	0.38 ± 0.20^£,§^	1.23 ± 0.26^a,^*	0.74 ± 0.55*^£^	0.25 ± 0.20^£,§^		
**Two-way ANOVA**													
**Parameters**		**Time**	**Diet**	**Time** × **Diet**	**Time**					**Diet**			
					**0** **h**	**4** **h**	**24** **h**	**48** **h**	**72** **h**	**NTRP**	**CTRL**	**TRP 13**	**TRP 17**
Thrombocytes		<0.001	ns	ns	A	B	AB	A	A	—	—	—	—
Lymphocytes		<0.001	0.005	ns	A	B	B	A	A	AB	A	B	B
Monocytes		<0.001	ns	0.001	B	B	B	B	A	—	—	—	—
Neutrophils		<0.001	ns	0.004	C	BC	A	A	B	—	—	—	—

Values are presented as means ± SD (n = 9 for time 0 h and n = 6 for the remaining times). P-values from two-way ANOVA (p ≤ 0.05). If interaction was significant, Tukey post hoc test was used to identify differences in the experimental treatments. Different lowercase letters stand for significant differences among dietary treatments for the same time while different symbols stand for significant differences between times for the same diet. Different capital letters indicate differences in time regardless diets or among diets regardless time.

#### Analysis of the peritoneal leucocytes responses

Total and differential peritoneal leucocytes counts were only performed in infected fish in order to assess cell migration dynamics to the inflammation site following bacterial injection, and are presented in Table 8. Fish fed NTRP presented lower concentration of peritoneal leucocytes relative to fish fed CTRL while both deficiency and supplementation presented lower macrophages concentration relative to fish fed CTRL, regardless time (Table 8). This results display the occurrence of a weaker local inflammatory response after the intraperitoneal infection with *Phdp* in fish fed with all dietary treatments compared to the control diet.

**Table 8:** Absolute values of peritoneal total leucocytes, as well as lymphocytes, macrophages and neutrophils of European seabass fed dietary treatments at 4, 24, 48 and 72 h after peritoneal injection with *Phdp*.

Parameters		Dietary treatments							
		NTRP				CTRL			
		4 h	24 h	48 h	72 h	4 h	24 h	48 h	72 h
Leucocytes	(×10^4^ µl^−1^)	9.89 ± 4.85	8.95 ± 4.95	7.83 ± 2.56	8.26 ± 2.91	11.51 ± 3.36	10.33 ± 4.60	13.63 ± 6.20	13.83 ± 8.04
Lymphocytes		3.78 ± 1.44	3.31 ± 2.16	3.19 ± 1.26	2.93 ± 1.62	2.96 ± 122	3.16 ± 1.68	5.17 ± 2.84	4.45 ± 2.13
Macrophages		3.23 ± 1.88	3.48 ± 1.72	2.72 ± 1.06	3.40 ± 1.10	5.24 ± 1.45	4.68 ± 2.48	5.29 ± 2.27	5.49 ± 4.00
Neutrophils		2.88 ± 1.65	2.16 ± 1.16	1.91 ± 0.60	1.98 ± 1.17	1.89 ± 0.78	2.3 ± 1.04	3.18 ± 1.32	4.13 ± 2.95
**Parameters**		**Dietary treatments**							
		**TRP 13**				**TRP 17**			
		**4** **h**	**24** **h**	**48** **h**	**72** **h**	**4** **h**	**24** **h**	**48** **h**	**72** **h**
Leucocytes	(×10^4^ µl^−1^)	8.71 ± 4.97	8.90 ± 2.77	9.88 ± 4.70	8.98 ± 7.27	10.95 ± 2.37	10.70 ± 4.42	9.35 ± 3.37	10.36 ± 4.71
Lymphocytes		3.13 ± 1.24	2.27 ± 0.68	3.58 ± 2.19	3.55 ± 3.06	4.73 ± 1.21	4.01 ± 1.46	3.55 ± 1.42	3.64 ± 1.53
Macrophages		3.49 ± 1.67	4.12 ± 1.48	4.38 ± 1.58	2.28 ± 2.28	3.53 ± 0.95	3.85 ± 2.34	3.76 ± 1.53	3.27 ± 1.64
Neutrophils		3.44 ± 1.57	2.41 ± 0.90	3.00 ± 1.17	2.92 ± 1.06	2.50 ± 0.55	3.09 ± 1.49	2.08 ± 0.77	3.49 ± 2.13
**Two-way ANOVA**									
**Parameters**		**Time**	**Diet**	**Time × Diet**	**Diet**				
					**NTRP**	**CTRL**	**TRP 13**	**TRP 17**	
Leucocytes		ns	0.025	ns	B	A	AB	AB	
Lymphocytes		ns	ns	ns	—	—	—	—	
Macrophages		ns	0.002	ns	B	A	B	B	
Neutrophils		ns	ns	ns	—	—	—	—	

Values are presented as means ± SD (n = 6). P-values from two-way ANOVA (p ≤ 0.05). If interaction was significant, Tukey post hoc test was used to identify differences in the experimental treatments. Different capital letters indicate differences among diets regardless time.

#### Plasma humoral responses

Plasma humoral immune responses as well as cortisol levels are presented in Table 9. Higher lysozyme concentration was found in fish fed TRP 17 relative to those fed NTRP. A time effect was observed for plasma lysozyme concentration with lower values at 24 and 48 h relative to 0 and 72 h while peroxidase levels decreased at 4 h relative to 0 and 72 h regardless dietary treatments. Additionally, plasma bactericidal activity augmented at 24 h compared to 0 and 48 h regardless dietary treatments. Fish fed NTRP presented higher plasma cortisol levels compared to fish fed CTRL and TRP 17 at 48 h. At the same time (48 h) NTRP presented significantly higher cortisol concentration relative to 0 h.

**Table 9:** Plasma lysozyme, peroxidase, alternative complement pathway (expressed as ACH50) and bactericidal activities as well as cortisol of European seabass fed dietary treatments prior infection (0 h) and at 4, 24, 48 and 72 h after peritoneal injection with *Phdp*.

Parameters		Dietary treatments											
		NTRP					CTRL						
		0 h	4 h	24 h	48 h	72 h	0 h	4 h	24 h	48 h	72 h		
Lysozyme	(µg ml^−1^)	8.11 ± 3.90	7.42 ± 1.2	5.00 ± 1.71	4.41 ± 2,47	8.11 ± 5.56	7.86 ± 2.23	9.26 ± 2.42	5.11 ± 2.45	5.95 ± 2.90	7.86 ± 3.11		
Peroxidase	(units ml^−1^)	118.63 ± 19.73	93.86 ± 19.00	115.84 ± 32.05	128.53 ± 45.05	126.55 ± 27.64	123.71 ± 13.94	96.83 ± 13.82	118.89 ± 28.75	111.48 ± 29.16	113.83 ± 31.17		
ACH50	(units ml^−1^)	92.16 ± 26.76	120.85 ± 26.09	85.32 ± 34.77	94.64 ± 23.34	102.78 ± 17.87	66.74 ± 10.31	142.35 ± 24.57	96.15 ± 28.66	103.52 ± 26.17	100.60 ± 15.14		
Bactericidal activity	(%)	26.38 ± 11.86	30.08 ± 2.81	38.97 ± 7.58	27.06 ± 13.48	28.70 ± 8.20	28.32 ± 7.64	28.49 ± 8.96	43.81 ± 9.44	29.84 ± 4.39	32.04 ± 4.48		
Cortisol	(ng ml^−1^)	25.99 ± 4.40^£^	50.44 ± 12.37^£,^*	65.43 ± 8.23^£,^*	103.11 ± 68.18^a,^*	72.22 ± 22.52^£,^*	27.74 ± 3.09	59.24 ± 5.00	51.05 ± 14.29	45.76 ± 8.90^b^	53.13 ± 23.37		
**Parameters**		**Dietary treatments**											
		**TRP 13**					**TRP 17**						
		**0** **h**	**4** **h**	**24** **h**	**48** **h**	**72** **h**	**0** **h**	**4** **h**	**24** **h**	**48** **h**	**72** **h**		
Lysozyme	(µg ml^−1^)	8.39 ± 1.94	7.84 ± 3.66	6.23 ± 1.75	6.17 ± 41.7	8.96 ± 2.73	10.97 ± 3.36	11.44 ± 5.23	7.61 ± ± 1.84	5.50 ± 2.47	10.32 ± 3.4		
Peroxidase	(units ml^−1^)	135.49 ± 27.82	108.82 ± 25.98	115.62 ± 16.20	102.16 ± 32.27	87.33 ± 14.55	139.23 ± 29.92	103.03 ± 21.26	113.44 ± 10.43	114.16 ± 31.95	85.29 ± 28.00		
ACH50	(units ml^−1^)	86.84 ± 22.67	58.42 ± 0.00	92.74 ± 28.44	139.49 ± 22.53	89.57 ± 24.25	119.60 ± 30.88	81.12 ± 12.14	99.24 ± 23.844	108.52 ± 30.32	126.56 ± 31.02		
Bactericidal activity	(%)	23.76 ± 14.90	33.62 ± 14.35	35.67 ± 4.30	30.14 ± 6.65	34.74 ± 12.37	17.43 ± 11.36	34.40 ± 9.59	43.17 ± 15.5	28.92 ± 5.75	31.26 ± 12.13		
Cortisol	(ng ml^−1^)	37.00 ± 6.67	50.54 ± 11.27	44.43 ± 8.51	61.70 ± 10.89^a,b^	32.62 ± 11.10	43.60 ± 12.88	42.00 ± 6.53	66.16 ± 17.87	33.44 ± 9.15^b^	44.57 ± 17.03		
**Two-way ANOVA**													
**Parameters**		**Time**	**Diet**	**Time** × **Diet**	**Time**					**Diet**			
					**0** **h**	**4** **h**	**24** **h**	**48** **h**	**72** **h**	**NTRP**	**CTRL**	**TRP 13**	**TRP 17**
Lysozyme		<0.001	0.039	ns	A	AB	B	B	A	B	AB	AB	A
Peroxidase		0.006	ns	ns	A	B	AB	AB	A	—	—	—	—
ACH50		ns	ns	ns	—	—	—	—	—	—	—	—	—
Bactericidal activity		<0.001	ns	ns	B	AB	A	B	AB	—	—	—	—
Cortisol		<0.001	0.007	0.001	B	AB	A	A	AB	A	AB	B	AB

Values are presented as means ± SD (n = 9 for time 0 h and n = 6 for the remaining times). P-values from two-way ANOVA (p ≤ 0.05). If interaction was significant, Tukey post hoc test was used to identify differences in the experimental treatments. Different lowercase letters stand for significant differences among dietary treatments for the same time while different symbols stand for significant differences between times for the same diet. Different capital letters indicate differences in time regardless diets or among diets regardless time.

#### Head-kidney gene expression

To evaluate the expression of genes related to immune response and tryptophan metabolism role in the inflammatory response (Table 5), cDNA was isolated from head-kidneys collected from 6 fish from each group (3 per replicate).

Regardless diet, a clear peak in the response to infection was observed at 48 h with *il1β*, *cox* 2, *m*2*cr*, *c3zeta*, *mcsf1r1*, *hsp70*, *dicent* and *mif* up-regulation than those registered at all sampling times. Moreover, *cd8β* was up-regulated at 48 h relative to 0 h whereas, *casp3* presented higher expression at 48 h compared to 0 and 24 h and *afmid* presented increased values at 48 h relative to 0, 24 and 72 h (Table S2).

**Figure 2:**
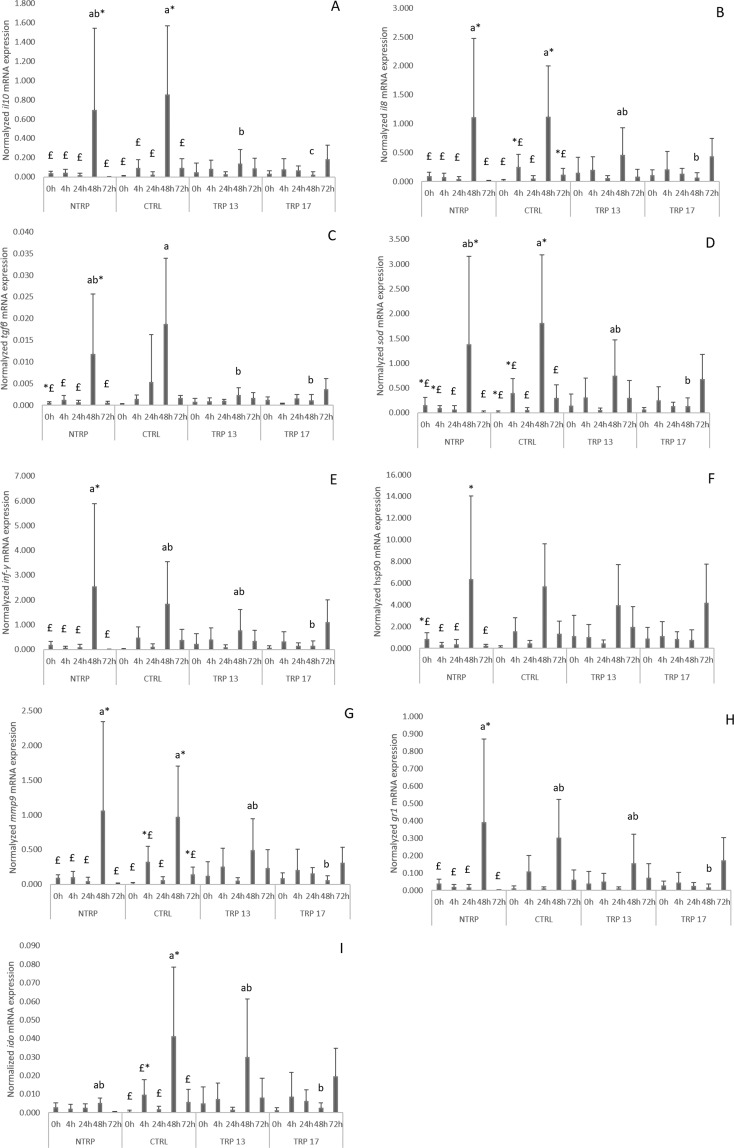
Quantitative expression of (**A**) interlukin-10, (**B**) interleukin-8; (**C**) transforming growth factor β, (**D**) superoxide dismutase, (**E**) interferon-γ, (**F**) heat shock protein 90, (**G**) metalloproteinase 9, (**H**) glucocorticoid receptor 1 and (**I**) indoleamine 2,3- dioxygenase genes in the head-kidney of European seabass fed dietary treatments prior infection, and at 4, 24, 48 and 72 h after peritoneal infection with *Phdp*. Values are presented as means ± SD (n = 6). P-values from two-way ANOVA (p ≤ 0.05). If interaction was significant, Tukey post hoc test was used to identify differences in the experimental treatments. Different lowercase letters stand for significant differences among dietary treatments for the same time while different symbols stand for significant differences between times for the same diet.

A time effect in fish fed NTRP at 48 h was observed as an increased expression of *il10* (Fig. 2A), *il8* (Fig. 2B), *ifn-γ* (Fig. 2E), *mmp9* (Fig. 2G) and *gr1* (Fig. 2H) compared to the remaining times was found, whereas those fish showed an enhanced expression for *tgfβ* (Fig. 2C) and *hsp90* (Fig. 2F) at the same time relative to 4, 24 and 72 h. Moreover, fish fed NTRP augmented *sod* mRNA expression at 48 h relative to 24 and 72 h. Fish fed the CTRL diet increased *il10* transcripts at 48 h relative to other times, whereas *il8* mRNA expression was higher at 48 h than at 24 h and *sod* and *mmp9* gene expression augmented at 48 h compared to 0 and 24 h. Fish fed the CTRL diet also presented an increase in *ido* 2 mRNA expression levels at 48 h relative to 0, 24 and 72 h.

A dietary effect was observed for *il*1*0* mRNA expression since higher levels were found in fish fed the CTRL diet compared to those fed TRP 13 and TRP 17, and also higher in specimens fed TRP 13 than in those fed TRP 17 at 48 h (Fig. 2A). Moreover, *il8* (Fig. 2B) *and mmp9* (Fig. 2G) transcripts decreased in fish fed TRP 17 compared to fish fed NTRP and CTRL at 48 h. Similarly, fish fed TRP 17 presented a decrease in *ifn-γ* (Fig. 2E) and *gr1* (Fig. 2H) transcripts relative to fish fed NTRP at the same time. Fish fed the CTRL diet presented an improved *tgfβ* mRNA expression compared to fish fed TRP 13 and TRP 17 at 48 h (Fig. 2C). Moreover, fish fed TRP 17 showed a decrease in *sod* (Fig. 2D) and *ido* 2 (Fig. 2I) transcripts compared to fish fed CTRL at 48 h. All data regarding gene expression are presented in Table S3 as Supplementary Data.

## Discussion

### Dietary tryptophan deficiency

As reviewed by Hoseini, *et al*.^4^, tryptophan deficiency is related to several physiological problems leading to scoliosis, opercula shortage and interference in mineral metabolism besides implications on fish growth. Despite these physiological signs were not reported in farmed fish, under stressful farming conditions tryptophan deficiency may occur due to an increase in tryptophan requirement^2,21^. Moreover, studies on the causative effect of a tryptophan deficient diet in fish immune mechanisms are scarce. In the present work no changes on growth performance, cell responses, cell-mediated mechanisms and gene expression were observed after a 4 weeks feeding period in fish fed the tryptophan deficient diet. It is therefore suggested that the level of tryptophan deficiency here tested may not have been low enough to impair physiological responses and growth during standardised (i.e. non-stressful) rearing conditions, a fact that could be linked to an overestimated tryptophan requirement for this species. However, it has been reported that free tryptophan concentration in plasma decreases during inflammation^2,22^ and the modulatory role of tryptophan under stressful events^23^ brings some awareness to its importance during the first steps of infection. In this context, and despite no differences were observed in the seabass immune status after 4 weeks of feeding, fish fed the NTRP diet presented a clear monocytopenia at 24 h post infection. A lower number of total leucocytes and macrophages were also found in the inflamed peritoneal cavity of fish fed NTRP relative to those fed CTRL. A decrease in the numbers of circulating B-lymphocytes contrasted the augmentation of peripheral neutrophils in several fish species as result of exposure to a variety of different stressors (e.g. transport, anoxia, social conflict, handling, injection, crowding)^24–26^. Moreover, Engelsma, *et al*.^27^ described that the lack of response observed in lymphocytes and neutrophils, as observed in this study, could be related to the response to an acute stress, as infection^27^. The observed drop in the total peripheral leucocyte population in the present study, and specifically the monocyte response, could also be related to the increase of plasma cortisol concentration at 48 h. In fact, a trend to present higher glucocorticoid receptor transcripts (*gr1*) was observed at 48 h. Cortisol capacity to modulate immune defence mechanisms in fish was already documented^4^, and its inhibitory capacity was observed in monocytes/macrophages cell line in rainbow trout (*Oncorhynchus mykiss*) reducing its proliferation^28^. Likewise, cortisol‐induced apoptosis was found in silver seabream (*Sparus sarba*) macrophages, and Atlantic salmon (*Salmo salar*) macrophages isolated from stressed fish^29^. Moreover, recognizing the antioxidant potential of tryptophan, mainly due the action of its metabolites (e.g. melatonin, 5-hydroxytryptophan, indole-3-acetic acid, 3-hydroxyanthranilic acid, 3-hydroxykynurenine)^4^, a dietary deficient scenario may have led to a lack of antioxidant signals ultimately inhibiting cell response. In the present study, the immune tolerance signal observed in infected fish translated in a decreased disease resistance against *Phdp*. Although no effects were observed in fish immune status, in an immune challenge scenario, when tryptophan requirements are increased and tryptophan is deficient in the diet, the possible lack of antioxidant signals and the visible increase of cortisol levels could have potentiated immunosuppressive mechanisms that impaired leucocytes response to infection and ultimately increased fish susceptibility to a bacterial pathogen.

### Dietary tryptophan supplementation

The tryptophan role in immune tolerance also adds a practical perspective to modern animal production counteracting the deleterious effect of an aggravated and uncontrolled immune response. Few available data were found concerning the effects of tryptophan dietary supplementation on immune mechanisms. Increasing dietary tryptophan levels improved anti-oxidative state and expression of genes related to immunity and amino acid transport in the ileal mucosa of broiler chickens^30^. In fish, previous works demonstrated that tryptophan supplementation (i.e. 0.5% DM) was not able to improve the European seabass^31^ nor Persian sturgeon (*Acipenser persicus*)^32^ immune status (cellular and plasma humoral parameters) after a 15 days feeding period. In accordance, the present study showed no significant changes in all the parameters tested with exception of plasma cortisol levels which increased with the highest tryptophan supplementation level tested (TRP 30) and *dicent* expression, an antimicrobial peptide, which was down-regulated in the HK of fish fed both supplemented diets. Tryptophan supplementation is known to stimulate cortisol production in unstressed fish^33^. Despite 5-HT levels were not verified in this study, in both teleost and mammals, the augmentation of tryptophan intake may increase its uptake in the brain where it is exponentially converted to 5-HT^34^, that can both stimulate or inhibit the adrenocorticotropic hormone (ACTH) production and thereby increase or inhibit cortisol production according to the stress situation^35^. The same was verified in juvenile rainbow trout when tryptophan was supplemented in diets of non-stressed fish increasing plasma cortisol levels while the opposite effect was observed in stressed groups^35^. Moreover, in common carp, *Cyprinus carpio*, juveniles cortisol was found increased when fed a 0.6% tryptophan supplemented diet in a pre-osmotic stress while mitigation the effect of the stress^36^.

In the present study, changes in blood leucocyte numbers were observed when inflammatory mechanisms were activated by *Phdp i*.*p*. injection. For instance, a significant reduction in the concentration of lymphocytes and monocytes and an increase in the numbers of neutrophils were recorded. Also, fish fed both supplemented diets showed a decrease in the migration of macrophages to the inflammatory focus. Engelsma, *et al*.^27^ described a similar phagocyte response for common carp (*Cyprinus carpio L*.) upon an acute stress as a reduction in the numbers of lymphocytes and monocytes was observed, in contrast to the increase in neutrophils numbers. Since neutrophils, as well as macrophages, are the primary line of defence, a prolongation of neutrophils lifespan together with an increase in the number of circulating functional neutrophils could be instrumental for the survival of an organism in acute stress situations and infection^27^. Similarly, lymphocytes and monocytes/macrophages response was modulated in European seabass stimulated with inactivated *Phdp* after a 15 days feeding period, showing a decrease in blood phagocytes numbers and restrained cell recruitment to the inflammatory peritoneal cavity^31^. In mice, dietary tryptophan supplementation was able to alleviate the inflammatory responses and in consequence, increase the reproductive performance of Pseudorabies virus-challenged female mice^37^.

Taking into consideration the enhanced cortisol concentration resulting from the 4 weeks feeding period described above, the cortisol role in the suppression of immune function^15^ may explain the lymphocyte and monocytes/macrophages response observed during an infection episode. In fact, this cell decrease was accompanied by a reduction of mRNA expression levels of pro-inflammatory cytokines (i.e. il1β, il8 and tgfβ), matrix metallopeptidase 9 (MMP9), an enzyme involved in the degradation of the extracellular matrix in processes such as cell migration and SOD, an enzyme involved in the antioxidant defence. Additionally, *ifn-γ* transcripts showed a trend to be diminished suggesting some lack of cell activation upon infection^38^. IFN-γ biological role is not fully elucidated yet, but it seems to present similar functions to mammalian IFN-γ^39^ being produced by natural killer cells (NK cells) and T lymphocytes in response to interleukin-12 (IL-12), IL-18, mitogens or antigens^40^.

The pathway of tryptophan catabolism present in macrophages and mediated by IDO, may also partly explain the lymphocyte and monocytes/macrophages decrease observed during infection. IFN-γ-induced IDO expression in monocytes leads to extracellular tryptophan depletion reducing its availability to microbial biosynthesis^9^. Additionally, through the kynurenine pathway, IDO is responsible for regulating T-cell function through metabolites such as 3-hydroxykynurenine, 3-hydroxyanthranilic acid and quinolinic acid. The same metabolites set up a protective system acting locally to the removal of superoxide radicals modulating oxidative status^10^, thus creating conditions that favour immune suppression and tolerance. Surprisingly, mRNA expression of *ido 2* was found down-regulated in a tryptophan supplementation scenario at 48 h relative to the control diet (CTRL). This could be related to the lower monocyte/macrophages proliferation and migration to the inflammatory site and since *ido 2* expression levels are necessarily associated to immune activation, its down-regulation seems to be in accordance with the generally suppressed immune response and to the drop in *ifn-γ* transcripts, since IFN-γ up-regulates IDO activity in macrophages^41^ and several cell lines^42^.

The overall results indicate that tryptophan supplementation seems to prime immune suppression and tolerance signals, in the highest supplementation level tested, most likely via higher plasma cortisol concentration observed after 4 weeks of feeding. While Hoseini and Hosseini^36^ discuss that tryptophan supplementation could enhance osmotic shock tolerance due to its immune tolerance role, our results shown in fact, a tendency for a higher disease susceptibility was observed when tryptophan was supplemented at the highest level, while no differences were observed regarding the control diet and the mid-term supplementation level.

In conclusion, results from the present study suggest that both dietary tryptophan deficiency and supplementation may compromise the inflammatory mechanisms and disease resistance. In a tryptophan deficiency scenario seabass immune status was not altered but in response to an inflammatory insult, plasma cortisol levels were significantly increased and consequently, the immune cell response was compromised, finally weakening fish disease resistance to *Phdp*. On the other hand, when dietary tryptophan is offered 26% above requirement level, cortisol levels rise despite no additional stress factor is inflicted on fish. Moreover, in response to bacterial infection, a decrease in lymphocytes, monocytes/macrophages and several immune-related genes is observed, eventually compromising at some level fish disease resistance.

## Material and Methods

### Experimental design

**Figure 3:**
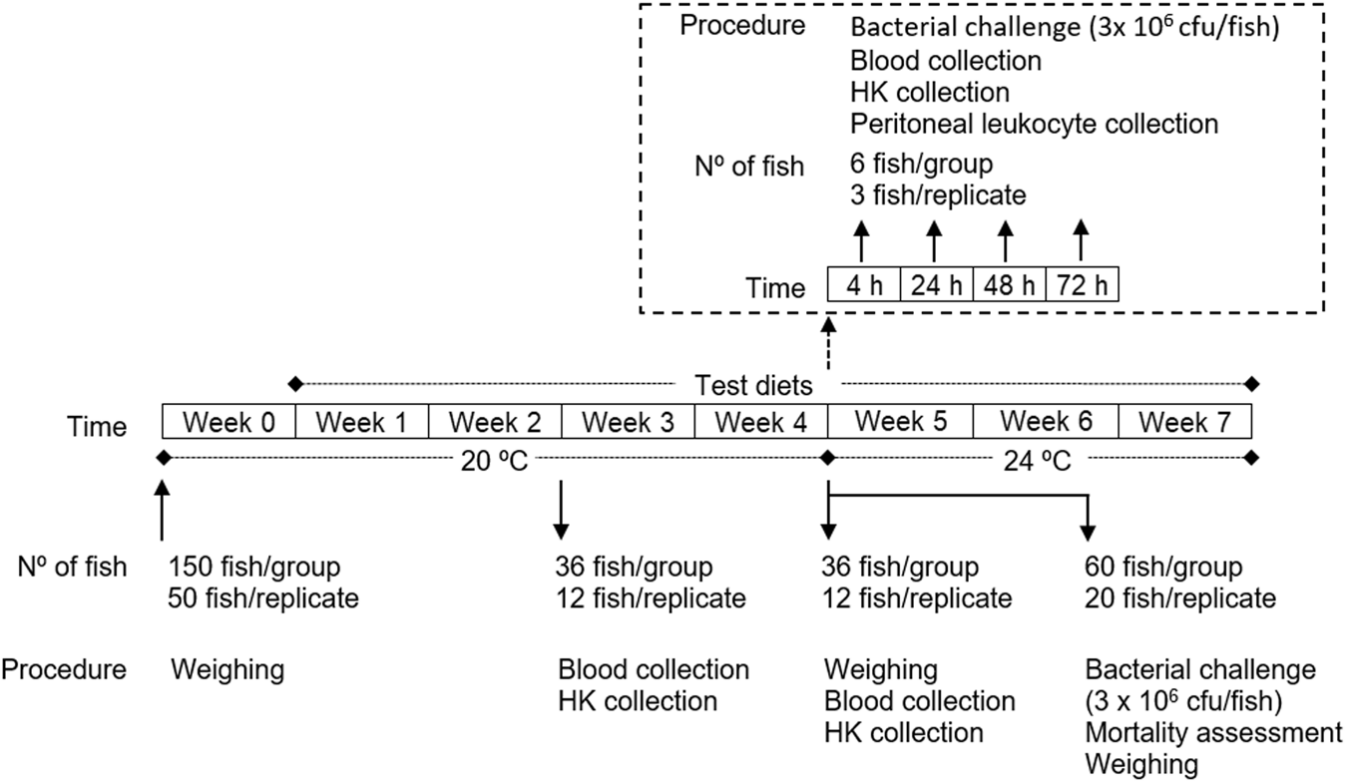
Experimental design.

European seabass juveniles were acquired to a certificated hatchery (MARESA, Spain) and kept in quarantine for two weeks at the Instituto de Investigação e Inovação em Saúde (i3S; University of Porto, Portugal) fish holding facilities under the culture conditions similar to Machado, *et al*.^43^ and described below. After this period, fish were weighed (Table 1) and randomly distributed into 12 tanks (200 L; 4 groups with 3 replicates of 50 fish each) of a recirculation seawater system in which O2 saturation (7.56 ± 0.24 mg/L), salinity (35 ppt) and photoperiod (10 h dark: 14 h light) were kept unchanged throughout the experiment (Fig. 3). Temperature was maintained at 20 ± 0.5 °C during the 4 weeks of feeding with the test diets and then increased to 24 ± 0.5 °C during the challenge period to mimic the temperature increase typical of piscine outbreaks. Ammonium and nitrite levels were kept below 0.025 and 0.3 mg L^−1^, respectively. The experiment was started by feeding of each group with the respective feed, 3 times a day at an average ration of 2.5% biomass per day. At 2 and 4 weeks of feeding, 36 fish from each group (12 per replicate) were euthanized by an overdose of anaesthetic (2-phenoxyethanol; Merck, ref. 807291, Germany), weighed, and collected blood and head kidney samples. Also at four weeks, fish that were not sampled (78 per group, 26 per replicate) were infected intraperitoneally (*i*.*p*.) with 100 μl of a *Phdp* suspension (3 × 10^7^ cfu ml^−1^). Of these, 60 fish per group (20 per replicate) were placed back in their tanks, fed according to the previous regime and mortality recorded for 3 weeks. After euthanasia of the moribund fish, the presence of *Phdp* in the head-kidney was checked by growing on tryptic soy agar supplemented with NaCl to a final concentration of 2% (w/v) (TSB-2) plates. The remaining infected fish (6 per group, 3 per replicate) were re-allocated in a similar recirculation system according to dietary treatment and 6 fish per group were euthanized at 4, 24, 48 and 72 hours post-infection (time-course) and blood, head-kidney and peritoneal exudates sampled from each fish.

The experiments were approved by the i3S Animal Welfare Committee and carried out in a registered installation (Licence Number 0421/000/000/2018) and performed by trained scientists in full compliance with national rules and following the European Directive 2010/63/EU of the European Parliament and the European Union Council on the protection of animals used for scientific purposes.

### Experimental diets

Four plant protein-based diets (Table 10) with inclusion of fish soluble protein concentrate (5%) for better palatability, were formulated and manufactured by Sparos Lda. (Olhão, Portugal). The NCTRL was formulated to include an indispensable AA concentration meeting the ideal pattern estimated for European seabass^44^ but deficient in tryptophan. The three other diets were identical to the NCTRL diet but supplemented with graded levels of L-tryptophan at 0.08, 0.13 and 0.17% of feed. After AA analysis NCTRL presented 17.14% less tryptophan in than CTRL, while tryptophan supplementation led to 2.28 and 25.71% above CTRL (TRP13 and TRP17, respectively). The supplementation levels were chosen according to previous works^31,45^ in order to test the effects of tryptophan deficiency, a slightly and more practical supplementation and a higher supplementation level.

**Table 10:** Ingredients and chemical composition of the experimental diets.

Ingredients	NTRP	CTRL	TRP 13	TRP 17
	%			
Fish soluble protein concentrate 90^a^	5.00	5.00	5.00	5.00
Fish gelatin^b^	2.00	2.00	2.00	2.00
Soy protein concentrate^c^	25.00	25.00	25.00	25.00
Pea protein concentrate^d^	6.00	6.00	6.00	6.00
Wheat gluten^e^	10.00	10.00	10.00	10.00
Corn gluten^f^	15.00	15.00	15.00	15.00
Wheat meal^g^	15.00	15.00	15.00	15.00
Fish oil^h^	15.90	15.90	15.90	15.90
Vitamin and mineral premix^i^	1.00	1.00	1.00	1.00
Soy lecithin^j^	1.00	1.00	1.00	1.00
Antioxidant^k^	0.20	0.20	0.20	0.20
Sodium propionate^l^	0.10	0.10	0.10	0.10
Monocalcium phosphate^m^	3.00	3.00	3.00	3.00
L-Lysine^n^	0.60	0.60	0.60	0.60
L-Tryptophan^o^	**0.00**	**0.08**	**0.13**	**0.17**
DL-Methionine^p^	0.20	0.20	0.20	0.20
Total	100.00	100.08	100.13	100.17
Pellet size, mm	1.50	1.50	1.50	1.50
**Proximative analyses (% dry weight)**				
Dry mater (%)	95.65	94.08	94.49	93.96
Ash (%)	5.84	5.47	5.50	5.55
Protein (%)	51.02	46.67	47.65	47.85
Fat (%)	19.58	19.03	18.38	18.39
Energy (kJ/g)	22.71	22.09	22.32	22.16
P (%)	0.94	0.66	0.66	0.90

^a^CPSP 90: 82.6% crude protein (CP), 9.6% crude fat (CF), Sopropêche, France. ^b^Fish gelatin: 88% CP, 0.1% CF, LAPI Gelatine SPA, Italy. ^c^Soycomil P: 63% CP, 0.8% CF, ADM, The Netherlands; ^d^NUTRALYS F85F: 78% CP, 1% CF, ROQUETTE Frères, France. ^e^VITAL: 83.7% CP, 1.6% CF, ROQUETTE Frères, France. ^f^Corn gluten meal: 61% CP, 6% CF, COPAM, Portugal. ^g^Wheat meal: 10.2% CP; 1.2% CF, Casa Lanchinha, Portugal. ^h^SAVINOR UTS, Portugal. ^i^PREMIX Lda, Portugal: Vitamins (IU or mg/kg diet): DL-alpha tocopherol acetate, 100 mg; sodium menadione bisulphate, 25 mg; retinyl acetate, 20000 IU; DL-cholecalciferol, 2000 IU; thiamin, 30 mg; riboflavin, 30 mg; pyridoxine, 20 mg; cyanocobalamin, 0.1 mg; nicotinic acid, 200 mg; folic acid, 15 mg; ascorbic acid, 500 mg; inositol, 500 mg; biotin, 3 mg; calcium panthotenate, 100 mg; choline chloride, 1000 mg, betaine, 500 mg. Minerals (g or mg/kg diet): copper sulphate, 9 mg; ferric sulphate, 6 mg; potassium iodide, 0.5 mg; manganese oxide, 9.6 mg; sodium selenite, 0.01 mg; zinc sulphate, 7.5 mg; sodium chloride, 400 mg; excipient wheat middlings. ^j^Lecico P700IPM, LECICO GmbH, Germany. ^k^Paramega PX, Kemin Europe NV, Belgium. ^l^PREMIX Lda., Portugal. ^m^MCP: 22% phosphorus, 16% calcium, Fosfitalia, Italy. ^n^Lysine HCl 99%, Ajinomoto Eurolysine SAS, France.

^o^L-Tryptophan 98%, Ajinomoto Eurolysine SAS, France. ^p^DL-Methionine for Aquaculture: 99% Methionine, Evonik Nutrition & Care GmbH, Germany

All diets were manufactured and AA content analysed as described by Machado, *et al*.^43^. Formulation, proximate analysis and AA profile of the experimental diets is presented in Tables 10 and 11, respectively. Tryptophan was determined by HPLC, after alkaline hydrolysis (Silliker Portugal, S.A.).

**Table 11:** Amino acid composition (g AA/100 g DW) of experimental diets.

Amino acids	NTRP	CTRL	TRP 13	TRP 17
Arginine	3.00	2.52	2.61	2.70
Histidine	1.09	0.86	0.92	0.98
Lysine	2.69	2.31	2.35	2.37
Threonine	2.05	1.9	1.75	1.57
Methionine	0.82	0.75	0.77	0.80
Cysteine	0.68	0.70	0.65	0.67
Methionine + Cysteine	1.50	1.44	1.42	1.47
Phenylalanine	2.59	2.2	2.32	2.24
Phenylalanine + Tyrosine	4.13	3.57	3.74	3.64
Taurine	0.02	0.02	0.03	0.02
Aspartic acid + Asparagine	4.44	3.96	3.86	3.96
Glutamic acid + Glutamine	11.7	10.2	10.3	10.3
Alanine	2.77	2.45	2.45	2.47
Glycine	2.67	2.27	2.24	2.26
Proline	5.63	4.12	4.66	4.94
Serine	2.79	2.57	2.53	2.51
Valine	1.85	1.37	1.61	1.59
Leucine	4.81	4.56	4.31	4.29
Isoleucine	1.91	1.78	1.59	1.57
Tyrosine	1.54	1.37	1.42	1.4
**Tryptophan**	**0.29** (20.68% below CTRL)	**0.35**	**0.36** (2.28% above CTRL)	**0.44** (25.71% above CTRL)

### Sample collection

#### Blood collection

Blood was collected from the caudal vessels with heparinized syringes one part being used for hematological analysis and the remain centrifuged at 10000 × *g* 10 min at 4 °C and the plasma collected, frozen on dry ice and stored at −80 °C.

Of the fish sampled at 2 and 4 weeks (36 fish per group; 12 per replicate), 9 fish from each group (3 per replicate) were used per time point for the hematological analysis. For the assessment of innate humoral immune response, plasma from all sampled fish were used and pooled every 3 individuals (12 pools per treatment).

Of the fish sampled at 4, 24, 48 and 72 hours after bacterial infection (6 fish per group; 3 per replicate) the hematological analysis and the evaluation of the innate humoral immune response parameters were performed for each individual.

#### Head kidney collection

Head kidneys were harvested from the 9 fish sampled at 2 and 4 weeks and of all fish sampled at 4, 24, 48 and 72 hours after infection. After harvesting, the kidneys were immediately frozen on dry ice and stored at −80 °C until processed for gene expression analysis.

#### Peritoneal exudates collection

Peritoneal cells were only sampled from fish sampled at 4, 24, 48 and 72 hours post-infection (time-course), according to the procedure described by Afonso, *et al*.^46^. The i.p. injected HBSS containing suspended cells were collected and total peritoneal leucocytes counts were performed with a hemocytometer.

### Analysis of haematological parameters

The haematological profile was conducted according to Machado, *et al*.^31^ and comprised of total white (WBC) and red (RBC) blood cells counts, haematocrit (Ht) and haemoglobin (Hb; SPINREACT kit, ref. 1001230, Spain). Subsequently, the mean corpuscular volume (MCV), mean corpuscular haemoglobin (MCH) and mean corpuscular haemoglobin concentration (MCHC) were calculated. Ht was not assessed in fish sampled at 4, 24 48 and 72 hours post-infection. Immediately after blood collection, blood smears were performed and absolute value (×10^4^ ml^−1^) of each cell type was calculated.

### Analysis of innate immune response parameters

Plasma lysozyme activity was measured as described by Costas, *et al*.^47^. Total peroxidase activity in plasma was evaluated following the procedure described by Quade and Roth^48^. Plasma bactericidal activity was determined following the method described by Graham and Secombes^49^ with modifications^31^ and the alternative complement pathway activity (ACH50) was evaluated as described by Sunyer and Tort^50^. Cortisol was assessed by an ELISA Kit (IBL International Gmbh, Hamburg, Germany) already validated for European seabass^45^, and following manufacturer’s instructions. All analyses were conducted in triplicates.

### Gene expression analysis

Total RNA isolation, first-strand cDNA synthesis, primers design and efficiency values and quantitative PCR assays were performed as described by Machado, *et al*.^43^. DNA amplification was carried out with specific primers (Table S3) for genes that have been selected for their involvement in immune responses and tryptophan metabolism (Table 5). Sequences encoding European seabass *c3zeta*, *mcsf1r1*, *cd8β*, *ido2* and *afmid* were identified after carrying out a search in the databases v1.0c seabass genome^51^ and designed as described in Machado, *et al*.^43^. Accession number, efficiency values, annealing temperature, product length and primers sequences are presented in Table S1. Melting curve analysis was also performed to verify that no primer dimers were amplified. The standard cycling conditions were 94 °C initial denaturation for 2 min, followed by 40 cycles of 94 °C denaturation for 30 s, primer annealing temperature (Table S3) for 30 s and 72 °C extension for 30 s. All reactions were carried out as technical duplicates. The expression of the target genes was normalized using the expression of European seabass *elongation factor 1*-*α* (*ef1α*).

### Analysis of the peritoneal leukocyte populations

After leukocyte collection as described in the 4.3.3 section, cytospin preparations were performed with a THARMAC Cellspin and stained as previously described for blood smears. Lymphocytes, macrophages and neutrophils in the peritoneal exudates were differentially counted, and the percentage of each cell type was established after counting a minimum of 200 cells per slide. Concentration (×10^4^ ml^−1^) of each leucocyte type was also calculated.

### Bacterial challenge

For the bacterial challenge, *Phdp*, strain PP3, isolated from yellowtail (*Seriola quinqueradiata*; Japan) by Dr Andrew C. Barnes (Marine Laboratory, Aberdeen, UK), was used. To prepare the inoculum for i.p. injection, 100 µL of stocked bacteria were cultured overnight at 22 °C on TSA-2. Exponentially growing bacteria were collected and re-suspended in sterile TSB-2 and adjusted to a final concentration of 3 × 10^7^ colony forming units (cfu) ml^−1^, as confirmed by plating the resulting cultures on TSA-2 plates and counting of cfu. Each fish was inoculated intraperitoneally with 100 µl (3 × 10^6^ cfu per fish) of the bacterial suspension.

### Data analysis

All results are expressed as mean ± standard deviation. Data was analysed for normality and homogeneity of variance and, transformed when necessary. All data expressed as percentage were arcsine transformed^52^. Data was analysed by two-way ANOVA, with time and diet as factors and followed by Tukey post-hoc test to identify differences in the experimental treatments (STATISTICA 12 for WINDOWS, P ≤ 0.05). Sampling point 4 weeks was used as time 0 h during time-course data analysis, as they represent unstimulated animal prior to infection. The Chi-square test was performed to identify differences on the cumulative mortality among dietary treatments.

## Supplementary information

Supplementaty file
