# Supplementary material for: Dietary tryptophan deficiency and its supplementation compromises inflammatory mechanisms and disease resistance in a teleost fish
Source: Sci Rep. 2019 May 22;9:7689. doi: 10.1038/s41598-019-44205-3 (PMC6531542; doi:10.1038/s41598-019-44205-3)
Supplement: Supplementary file 1 — Supplementaty file [file 41598_2019_44205_MOESM1_ESM.docx]

Dietary tryptophan deficiency and its supplementation compromises inflammatory mechanisms and disease resistance in a teleost fish

M. Machado^1,2,3,4*^, R. Azeredo^1,3^, A. Domingues^,2,4^, S. Fernandez-Boo^1^, L.E.C. Conceição^5^, J. Dias^5^ and B. Costas^1,3*^

^1^ Centro Interdisciplinar de Investigação Marinha e Ambiental (CIIMAR), Terminal de Cruzeiros do Porto de Leixões, Av. General Norton de Matos s/n, 4450-208 Matosinhos
Portugal.

^2^ Instituto de Investigação e Inovação em Saúde (i3S), Universidade do Porto, Rua Alfredo Allen, 208, 4200-135 Porto, Portugal.

^3^ Instituto de Ciências Biomédicas Abel Salazar (ICBAS-UP), Universidade do Porto, Rua de Jorge Viterbo Ferreira nº 228, 4050-313 Porto, Portugal.

^4^ Instituto de Biologia Molecular e Celular, Universidade do Porto, Rua Alfredo Allen, 208, 4200-135 Porto, Portugal.

^5^ Sparos Lda, Area Empresarial de Marim, Lote C, Olhão, Portugal.

***Corresponding authors:**

Centro Interdisciplinar de Investigação Marinha e Ambiental (CIIMAR), Terminal de Cruzeiros do Porto de Leixões, Avenida General Norton de Matos s/n, 4450-208 Matosinhos, Portugal.

Tel.: +351 223401850; fax: +351 223401838.

Email address: mcasimiro@ciimar.up.pt; bcostas@ciimar.up.pt

**Supplementary File**

**Table S1.** Quantitative expression of immune-related genes in the head-kidney of European seabass fed dietary treatments during 2 and 4 weeks.

| Parameters | |  | Dietary treatments | | | | | | | | | | | | | | | | | | | | | | | | | | |
| --- | --- | --- | --- | --- | --- | --- | --- | --- | --- | --- | --- | --- | --- | --- | --- | --- | --- | --- | --- | --- | --- | --- | --- | --- | --- | --- | --- | --- | --- |
|  |  |  | NTRP | | | | | |  | CTRL | | | | | |  | TRP 13 | | | | | |  | TRP 17 | | | | | |
|  |  |  | 2 weeks | | | 4 weeks | | |  | 2 weeks | | | 4 weeks | | |  | 2 weeks | | | 4 weeks | | |  | 2 weeks | | | 4 weeks | | |
| *il1β* | Normalized mRNA expression |  | 0.109 | ± | 0.110 | 0.049 | ± | 0.031 |  | 0.073 | ± | 0.038 | 0.007 | ± | 0.007 |  | 0.061 | ± | 0.083 | 0.020 | ± | 0.030 |  | 0.095 | ± | 0.074 | 0.040 | ± | 0.037 |
| *il10* |  |  | 0.083 | ± | 0.094 | 0.038 | ± | 0.022 |  | 0.075 | ± | 0.061 | 0.008 | ± | 0.005 |  | 0.085 | ± | 0.105 | 0.050 | ± | 0.091 |  | 0.061 | ± | 0.049 | 0.034 | ± | 0.031 |
| *il8* |  |  | 0.252 | ± | 0.304 | 0.095 | ± | 0.061 |  | 0.207 | ± | 0.170 | 0.017 | ± | 0.017 |  | 0.141 | ± | 0.123 | 0.149 | ± | 0.274 |  | 0.145 | ± | 0.105 | 0.108 | ± | 0.097 |
| *tgfβ* |  |  | 0.001 | ± | 0.001 | 0.001 | ± | 0.000 |  | 0.001 | ± | 0.000 | 0.000 | ± | 0.000 |  | 0.001 | ± | 0.001 | 0.001 | ± | 0.001 |  | 0.000 | ± | 0.000 | 0.001 | ± | 0.001 |
| *sod* |  |  | 0.255 | ± | 0.278 | 0.154 | ± | 0.160 |  | 0.275 | ± | 0.280 | 0.021 | ± | 0.015 |  | 0.214 | ± | 0.249 | 0.145 | ± | 0.234 |  | 0.226 | ± | 0.148 | 0.067 | ± | 0.037 |
| *cox 2* |  |  | 3.403 | ± | 3.583 | 1.291 | ± | 0.597 |  | 3.241 | ± | 2.100 | 0.200 | ± | 0.158 |  | 3.432 | ± | 3.625 | 1.531 | ± | 2.800 |  | 2.438 | ± | 1.785 | 0.974 | ± | 0.949 |
| *m2cr* |  |  | 0.010 | ± | 0.013 | 0.001 | ± | 0.001 |  | 0.005 | ± | 0.004 | 0.000 | ± | 0.000 |  | 0.004 | ± | 0.005 | 0.002 | ± | 0.004 |  | 0.003 | ± | 0.002 | 0.004 | ± | 0.003 |
| *ifn-γ* |  |  | 0.631 | ± | 0.778 | 0.186 | ± | 0.137 |  | 0.401 | ± | 0.388 | 0.022 | ± | 0.023 |  | 0.394 | ± | 0.462 | 0.224 | ± | 0.422 |  | 0.395 | ± | 0.262 | 0.082 | ± | 0.071 |
| *c3zeta* |  |  | 0.006 | ± | 0.008 | 0.001 | ± | 0.001 |  | 0.004 | ± | 0.003 | 0.000 | ± | 0.000 |  | 0.003 | ± | 0.003 | 0.002 | ± | 0.002 |  | 0.002 | ± | 0.001 | 0.001 | ± | 0.001 |
| *mcsf1r1* |  |  | 0.003 | ± | 0.002 | 0.006 | ± | 0.005 |  | 0.006 | ± | 0.007 | 0.002 | ± | 0.001 |  | 0.003 | ± | 0.004 | 0.007 | ± | 0.010 |  | 0.002 | ± | 0.002 | 0.007 | ± | 0.008 |
| *cd8β* |  |  | 0.044 | ± | 0.061 | 0.006 | ± | 0.006 |  | 0.037 | ± | 0.025 | 0.000 | ± | 0.000 |  | 0.032 | ± | 0.042 | 0.001 | ± | 0.002 |  | 0.018 | ± | 0.012 | 0.032 | ± | 0.064 |
| *hsp70* |  |  | 0.044 | ± | 0.060 | 0.018 | ± | 0.011 |  | 0.038 | ± | 0.034 | 0.004 | ± | 0.002 |  | 0.030 | ± | 0.032 | 0.013 | ± | 0.023 |  | 0.033 | ± | 0.023 | 0.016 | ± | 0.015 |
| *hsp90* |  |  | 1.768 | ± | 1.876 | 0.847 | ± | 0.582 |  | 1.890 | ± | 1.486 | 0.140 | ± | 0.077 |  | 1.786 | ± | 1.804 | 1.113 | ± | 1.931 |  | 1.529 | ± | 1.106 | 0.902 | ± | 1.005 |
| *mmp9* |  |  | 0.274 | ± | 0.393 | 0.092 | ± | 0.047 |  | 0.205 | ± | 0.188 | 0.014 | ± | 0.011 |  | 0.199 | ± | 0.222 | 0.120 | ± | 0.204 |  | 0.154 | ± | 0.129 | 0.087 | ± | 0.077 |
| *dicent* |  |  | 0.491 | ± | 0.440^a^ | 0.107 | ± | 0.044 |  | 0.518 | ± | 0.348^a^* | 0.035 | ± | 0.024 |  | 0.107 | ± | 0.078^b^ | 0.242 | ± | 0.406 |  | 0.070 | ± | 0.036^b^ | 0.179 | ± | 0.182 |
| *gr1* |  |  | 0.124 | ± | 0.153 | 0.039 | ± | 0.024 |  | 0.091 | ± | 0.051 | 0.012 | ± | 0.012 |  | 0.071 | ± | 0.069 | 0.040 | ± | 0.068 |  | 0.072 | ± | 0.041 | 0.028 | ± | 0.024 |
| *mif* |  |  | 0.009 | ± | 0.009 | 0.003 | ± | 0.002 |  | 0.007 | ± | 0.004 | 0.001 | ± | 0.000 |  | 0.006 | ± | 0.006 | 0.004 | ± | 0.008 |  | 0.004 | ± | 0.002 | 0.003 | ± | 0.003 |
| *casp3* |  |  | 0.026 |  | 0.034 | 0.008 | ± | 0.007 |  | 0.009 | ± | 0.007 | 0.001 | ± | 0.001 |  | 0.017 | ± | 0.019 | 0.019 |  | 0.034 |  | 0.019 | ± | 0.013 | 0.008 | ± | 0.004 |
| *ido2* |  |  | 0.010 | ± | 0.010 | 0.003 | ± | 0.002 |  | 0.006 | ± | 0.005 | 0.001 | ± | 0.001 |  | 0.005 | ± | 0.006 | 0.005 | ± | 0.009 |  | 0.004 | ± | 0.002 | 0.001 | ± | 0.001 |
| *afmid* |  |  | 0.020 | ± | 0.024 | 0.005 | ± | 0.004 |  | 0.017 | ± | 0.001 | 0.001 | ± | 0.001 |  | 0.013 | ± | 0.017 | 0.008 | ± | 0.006 |  | 0.008 | ± | 0.006 | 0.004 | ± | 0.005 |

| Two-way ANOVA | | |  |  |
| --- | --- | --- | --- | --- |
| Parameters | |  |  |  |
|  |  | Time | Diet | Time × Diet |
| *il1β* |  | 0.011 | ns | ns |
| *il10* |  | 0.046 | ns | ns |
| *il8* |  | ns | ns | ns |
| *tgfβ* |  | ns | ns | ns |
| *sod* |  | 0.027 | ns | ns |
| *cox 2* |  | 0.005 | ns | ns |
| *m2cr* |  | 0.046 | ns | ns |
| *ifn-γ* |  | 0.012 | ns | ns |
| *c3zeta* |  | 0.013 | ns | ns |
| *mcsf1r1* |  | ns | ns | ns |
| *cd8β* |  | ns | ns | ns |
| *hsp70* |  | 0.017 | ns | ns |
| *hsp90* |  | < 0.001 | ns | ns |
| *mmp9* |  | 0.049 | ns | ns |
| *dicent* |  | ns | ns | 0.014 |
| *gr1* |  | 0.009 | ns | ns |
| *mif* |  | 0.026 | ns | ns |
| *casp3* |  | ns | ns | ns |
| *ido2* |  | 0.033 | ns | ns |
| *afmid* |  | 0.023 | ns | ns |

Values are presented as means ± SD (n=6). P-values from two-way ANOVA (p ≤0.05). If interaction was significant, Tukey post hoc test was used to identify differences in the experimental treatments. Different lowercase letters stand for significant differences among dietary treatments for the same time while different symbols stands for significant differences between times for the same diet.

**Table S2.** Quantitative expression of immune-related genes in the head-kidney of European seabass fed dietary treatments prior infection (0 h), and at 4, 24, 48 and 72 h after peritoneal injection with *Phdp*.

| Parameters | |  | Dietary treatments | | | | | | | | | | | | | | | | | | | | | | | | | | | | | | |
| --- | --- | --- | --- | --- | --- | --- | --- | --- | --- | --- | --- | --- | --- | --- | --- | --- | --- | --- | --- | --- | --- | --- | --- | --- | --- | --- | --- | --- | --- | --- | --- | --- | --- |
|  |  |  | NTRP | | | | | | | | | | | | | | |  | CTRL | | | | | | | | | | | | | | |
|  |  |  | 0h | | | 4h | | | 24h | | | 48h | | | 72h | | |  | 0h | | | 4h | | | 24h | | | 48h | | | 72h | | |
| *il1β* | Normalized mRNA expression |  | 0.049 | ± | 0.031 | 0.032 | ± | 0.027 | 0.023 | ± | 0.030 | 0.477 | ± | 0.600 | 0.003 | ± | 0.004 |  | 0.007 | ± | 0.007 | 0.178 | ± | 0.149 | 0.028 | ± | 0.026 | 0.423 | ± | 0.369 | 0.080 | ± | 0.076 |
| *il10* |  |  | 0.038 | ± | 0.022^£^ | 0.045 | ± | 0.032^£^ | 0.018 | ± | 0.019^£^ | 0.697 | ± | 0.845^ab^* | 0.002 | ± | 0.003^£^ | | 0.008 | ± | 0.005^£^ | 0.096 | ± | 0.082^£^ | 0.026 | ± | 0.026^£^ | 0.856 | ± | 0.712^a^* | 0.096 | ± | 0.093^£^ |
| *il8* |  |  | 0.095 | ± | 0.061^£^ | 0.080 | ± | 0.062^£^ | 0.042 | ± | 0.046^£^ | 1.116 | ± | 1.362^a^* | 0.008 | ± | 0.008^£^ | | 0.017 | ± | 0.017^£^ | 0.249 | ± | 0.222^£^* | 0.051 | ± | 0.050^£^ | 1.118 | ± | 0.884^a^* | 0.122 | ± | 0.104^£^* |
| *tgfβ* |  |  | 0.001 | ± | 0.000^£^* | 0.001 | ± | 0.001^£^ | 0.001 | ± | 0.000^£^ | 0.012 | ± | 0.014^ab^* | 0.001 | ± | 0.000^£^ | | 0.000 | ± | 0.000 | 0.001 | ± | 0.001 | 0.005 | ± | 0.011 | 0.019 | ± | 0.015^a^ | 0.002 | ± | 0.001 |
| *sod* |  |  | 0.154 | ± | 0.160^£^* | 0.094 | ± | 0.051^£^* | 0.067 | ± | 0.077^£^ | 1.381 | ± | 1.777^ab^* | 0.021 | ± | 0.012^£^ | | 0.021 | ± | 0.015^£^* | 0.394 | ± | 0.293^£^* | 0.060 | ± | 0.040^£^ | 1.815 | ± | 1.369^a^* | 0.304 | ± | 0.264^£^ |
| *cox 2* |  |  | 1.291 | ± | 0.597 | 0.747 | ± | 0.506 | 0.680 | ± | 0.734 | 8.627 | ± | 8.198 | 7.915 | ± | 0.174 |  | 0.200 | ± | 0.158 | 2.708 | ± | 1.959 | 0.638 | ± | 0.601 | 8.595 | ± | 7.963 | 0.883 | ± | 0.460 |
| *m2cr* |  |  | 0.001 | ± | 0.001 | 0.005 | ± | 0.005 | 0.040 | ± | 0.089 | 0.131 | ± | 0.172 | 0.000 | ± | 0.000 |  | 0.000 | ± | 0.000 | 0.019 | ± | 0.019 | 0.000 | ± | 0.000 | 0.109 | ± | 0.082 | 0.004 | ± | 0.003 |
| *ifn-γ* |  |  | 0.186 | ± | 0.137^£^ | 0.075 | ± | 0.066^£^ | 0.090 | ± | 0.114^£^ | 2.547 | ± | 3.333^a^* | 0.009 | ± | 0.009^£^ | | 0.022 | ± | 0.023 | 0.481 | ± | 0.436 | 0.114 | ± | 0.126 | 1.853 | ± | 1.683^ab^ | 0.396 | ± | 0.413 |
| *c3zeta* |  |  | 0.001 | ± | 0.001 | 0.000 | ± | 0.000 | 0.000 | ± | 0.000 | 0.026 | ± | 0.038 | 0.000 | ± | 0.000 |  | 0.000 | ± | 0.000 | 0.002 | ± | 0.002 | 0.003 | ± | 0.006 | 0.047 | ± | 0.049 | 0.005 | ± | 0.008 |
| *mcsf1r1* |  |  | 0.006 | ± | 0.005 | 0.002 | ± | 0.003 | 0.002 | ± | 0.003 | 0.071 | ± | 0.094 | 0.000 | ± | 0.000 |  | 0.002 | ± | 0.001 | 0.009 | ± | 0.007 | 0.001 | ± | 0.001 | 0.050 | ± | 0.044 | 0.003 | ± | 0.003 |
| *cd8β* |  |  | 0.006 | ± | 0.006 | 0.058 | ± | 0.044 | 0.066 | ± | 0.071 | 0.619 | ± | 0.852 | 0.005 | ± | 0.004 |  | 0.000 | ± | 0.000 | 0.286 | ± | 0.269 | 0.078 | ± | 0.088 | 0.156 | ± | 0.160 | 0.184 | ± | 0.144 |
| *hsp70* |  |  | 0.018 | ± | 0.011^£^* | 0.012 | ± | 0.010^£^ | 0.007 | ± | 0.006^£^ | 0.433 | ± | 0.564* | 0.003 | ± | 0.002^£^ | | 0.004 | ± | 0.002 | 0.034 | ± | 0.033 | 0.013 | ± | 0.013 | 0.250 | ± | 0.195 | 0.045 | ± | 0.042 |
| *hsp90* |  |  | 0.847 | ± | 0.582^£^* | 0.338 | ± | 0.221^£^ | 0.380 | ± | 0.423^£^ | 6.390 | ± | 7.639* | 0.220 | ± | 0.148^£^ |  | 0.140 | ± | 0.077 | 1.556 | ± | 1.266 | 0.434 | ± | 0.295 | 5.706 | ± | 3.944 | 1.355 | ± | 1.128 |
| *mmp9* |  |  | 0.092 | ± | 0.047^£^ | 0.104 | ± | 0.080^£^ | 0.048 | ± | 0.057^£^ | 1.060 | ± | 1.282^a^* | 0.009 | ± | 0.009^£^ | | 0.014 | ± | 0.011^£^ | 0.325 | ± | 0.224^ab^ | 0.058 | ± | 0.054^£^ | 0.972 | ± | 0.728^a^* | 0.140 | ± | 0.110^£^* |
| *dicent* |  |  | 0.107 | ± | 0.044 | 0.118 | ± | 0.065 | 0.041 | ± | 0.055 | 2.240 | ± | 2.799 | 0.130 | ± | 0.116 |  | 0.035 | ± | 0.024 | 0.434 | ± | 0.221 | 0.050 | ± | 0.017 | 1.354 | ± | 0.923 | 0.108 | ± | 0.047 |
| *gr1* |  |  | 0.039 | ± | 0.024^£^ | 0.020 | ± | 0.014^£^ | 0.016 | ± | 0.018^£^ | 0.393 | ± | 0.479^a^* | 0.002 | ± | 0.002^£^ | | 0.012 | ± | 0.012 | 0.108 | ± | 0.093 | 0.011 | ± | 0.009 | 0.303 | ± | 0.219^ab^ | 0.062 | ± | 0.055 |
| *mif* |  |  | 0.003 | ± | 0.002 | 0.004 | ± | 0.005 | 0.001 | ± | 0.001 | 0.033 | ± | 0.043 | 0.013 | ± | 0.028 |  | 0.001 | ± | 0.000 | 0.015 | ± | 0.013 | 0.001 | ± | 0.001 | 0.054 | ± | 0.052 | 0.007 | ± | 0.007 |
| *casp3* |  |  | 0.008 | ± | 0.007 | 0.009 | ± | 0.010 | 0.006 | ± | 0.007 | 0.203 | ± | 0.288 | 0.001 | ± | 0.001 |  | 0.001 | ± | 0.001 | 0.055 | ± | 0.042 | 0.006 | ± | 0.009 | 0.074 | ± | 0.143 | 0.014 | ± | 0.019 |
| *ido2* |  |  | 0.003 | ± | 0.002 | 0.002 | ± | 0.002 | 0.003 | ± | 0.002 | 0.005 | ± | 0.003^ab^ | 0.000 | ± | 0.000 |  | 0.001 | ± | 0.001^£^ | 0.010 | ± | 0.008^£^* | 0.002 | ± | 0.002^£^ | 0.041 | ± | 0.037^a^ | 0.006 | ± | 0.007^£^ |
| *afmid* |  |  | 0.005 | ± | 0.004 | 0.004 | ± | 0.004 | 0.002 | ± | 0.002 | 0.055 | ± | 0.074 | 0.000 | ± | 0.000 |  | 0.001 | ± | 0.001 | 0.018 | ± | 0.013 | 0.002 | ± | 0.003 | 0.060 | ± | 0.054 | 0.007 | ± | 0.007 |

| Parameters | |  | Dietary treatments | | | | | | | | | | | | | | | | | | | | | | | | | | | | | | |
| --- | --- | --- | --- | --- | --- | --- | --- | --- | --- | --- | --- | --- | --- | --- | --- | --- | --- | --- | --- | --- | --- | --- | --- | --- | --- | --- | --- | --- | --- | --- | --- | --- | --- |
|  |  |  | TRP 13 | | | | | | | | | | | | | | |  | TRP 17 | | | | | | | | | | | | | | |
|  |  |  | 0h | | | 4h | | | 24h | | | 48h | | | 72h | | |  | 0h | | | 4h | | | 24h | | | 48h | | | 72h | | |
| *il1β* | Normalized mRNA expression |  | 0.020 | ± | 0.030 | 0.059 | ± | 0.073 | 0.018 | ± | 0.018 | 0.282 | ± | 0.325 | 0.086 | ± | 0.109 |  | 0.040 | ± | 0.037 | 0.107 | ± | 0.127 | 0.165 | ± | 0.259 | 0.022 | ± | 0.036 | 0.192 | ± | 0.171 |
| *il10* |  |  | 0.050 | ± | 0.091 | 0.081 | ± | 0.092 | 0.027 | ± | 0.029 | 0.140 | ± | 0.145^b^ | 0.088 | ± | 0.105 |  | 0.034 | ± | 0.031 | 0.079 | ± | 0.108 | 0.070 | ± | 0.044 | 0.026 | ± | 0.028^c^ | 0.184 | ± | 0.146 |
| *il8* |  |  | 0.149 | ± | 0.274 | 0.202 | ± | 0.222 | 0.056 | ± | 0.044 | 0.462 | ± | 0.467^ab^ | 0.087 | ± | 0.126 |  | 0.108 | ± | 0.097 | 0.210 | ± | 0.312 | 0.137 | ± | 0.090 | 0.070 | ± | 0.082^b^ | 0.436 | ± | 0.308 |
| *tgfβ* |  |  | 0.001 | ± | 0.001 | 0.001 | ± | 0.001 | 0.001 | ± | 0.000 | 0.002 | ± | 0.002^b^ | 0.002 | ± | 0.001 |  | 0.001 | ± | 0.001 | 0.000 | ± | 0.000 | 0.002 | ± | 0.001 | 0.001 | ± | 0.001^b^ | 0.004 | ± | 0.002 |
| *sod* |  |  | 0.145 | ± | 0.234 | 0.315 | ± | 0.381 | 0.056 | ± | 0.044 | 0.750 | ± | 0.719^ab^ | 0.306 | ± | 0.351 |  | 0.067 | ± | 0.037 | 0.257 | ± | 0.265 | 0.131 | ± | 0.081 | 0.140 | ± | 0.163^b^ | 0.681 | ± | 0.497 |
| *cox 2* |  |  | 1.531 | ± | 2.800 | 1.957 | ± | 2.003 | 0.512 | ± | 0.431 | 11.795 | ± | 7.027 | 1.455 | ± | 0.698 |  | 0.974 | ± | 0.949 | 2.035 | ± | 2.444 | 1.033 | ± | 0.842 | 3.941 | ± | 0.783 | 0.112 | ± | 3.397 |
| *m2cr* |  |  | 0.002 | ± | 0.004 | 0.010 | ± | 0.011 | 0.002 | ± | 0.002 | 0.066 | ± | 0.071 | 0.005 | ± | 0.006 |  | 0.004 | ± | 0.003 | 0.016 | ± | 0.020 | 0.007 | ± | 0.006 | 0.003 | ± | 0.003 | 0.028 | ± | 0.020 |
| *ifn-γ* |  |  | 0.224 | ± | 0.422 | 0.408 | ± | 0.458 | 0.103 | ± | 0.091 | 0.785 | ± | 0.836^ab^ | 0.351 | ± | 0.425 |  | 0.082 | ± | 0.071 | 0.325 | ± | 0.392 | 0.157 | ± | 0.112 | 0.149 | ± | 0.192^b^ | 1.102 | ± | 0.904 |
| *c3zeta* |  |  | 0.002 | ± | 0.002 | 0.004 | ± | 0.005 | 0.001 | ± | 0.001 | 0.018 | ± | 0.027 | 0.009 | ± | 0.015 |  | 0.001 | ± | 0.001 | 0.002 | ± | 0.001 | 0.005 | ± | 0.005 | 0.001 | ± | 0.001 | 0.009 | ± | 0.008 |
| *mcsf1r1* |  |  | 0.007 | ± | 0.010 | 0.008 | ± | 0.009 | 0.002 | ± | 0.002 | 0.048 | ± | 0.060 | 0.003 | ± | 0.004 |  | 0.007 | ± | 0.008 | 0.011 | ± | 0.018 | 0.006 | ± | 0.003 | 0.002 | ± | 0.004 | 0.024 | ± | 0.027 |
| *cd8β* |  |  | 0.001 | ± | 0.002 | 0.268 | ± | 0.314 | 0.014 | ± | 0.021 | 0.190 | ± | 0.167 | 0.206 | ± | 0.354 |  | 0.032 | ± | 0.064 | 0.246 | ± | 0.324 | 0.006 | ± | 0.005 | 0.030 | ± | 0.036 | 0.019 | ± | 0.016 |
| *hsp70* |  |  | 0.013 | ± | 0.023 | 0.036 | ± | 0.044 | 0.011 | ± | 0.007 | 0.175 | ± | 0.194 | 0.046 | ± | 0.048 |  | 0.016 | ± | 0.015 | 0.040 | ± | 0.061 | 0.025 | ± | 0.017 | 0.015 | ± | 0.016 | 0.112 | ± | 0.082 |
| *hsp90* |  |  | 1.113 | ± | 1.931 | 1.039 | ± | 1.154 | 0.441 | ± | 0.305 | 3.960 | ± | 3.749 | 1.955 | ± | 1.904 |  | 0.902 | ± | 1.005 | 1.136 | ± | 1.331 | 0.857 | ± | 0.673 | 0.785 | ± | 0.907 | 4.182 | ± | 3.557 |
| *mmp9* |  |  | 0.120 | ± | 0.204 | 0.252 | ± | 0.270 | 0.055 | ± | 0.043 | 0.493 | ± | 0.454^ab^ | 0.235 | ± | 0.264 |  | 0.087 | ± | 0.077 | 0.208 | ± | 0.295 | 0.157 | ± | 0.087 | 0.060 | ± | 0.064^b^ | 0.311 | ± | 0.222 |
| *dicent* |  |  | 0.242 | ± | 0.406 | 0.388 | ± | 0.364 | 0.058 | ± | 0.034 | 1.288 | ± | 1.269 | 0.121 | ± | 0.112 |  | 0.179 | ± | 0.182 | 0.250 | ± | 0.472 | 0.216 | ± | 0.141 | 0.260 | ± | 0.201 | 0.240 | ± | 0.197 |
| *gr1* |  |  | 0.040 | ± | 0.068 | 0.049 | ± | 0.048 | 0.010 | ± | 0.009 | 0.155 | ± | 0.168^ab^ | 0.073 | ± | 0.081 |  | 0.028 | ± | 0.024 | 0.045 | ± | 0.057 | 0.025 | ± | 0.021 | 0.018 | ± | 0.019^b^ | 0.172 | ± | 0.132 |
| *mif* |  |  | 0.004 | ± | 0.008 | 0.004 | ± | 0.003 | 0.001 | ± | 0.001 | 0.016 | ± | 0.017 | 0.006 | ± | 0.007 |  | 0.003 | ± | 0.003 | 0.006 | ± | 0.010 | 0.002 | ± | 0.002 | 0.003 | ± | 0.004 | 0.015 | ± | 0.013 |
| *casp3* |  |  | 0.019 | ± | 0.034 | 0.056 | ± | 0.086 | 0.005 | ± | 0.007 | 0.121 | ± | 0.145 | 0.067 |  | 0.090 |  | 0.008 | ± | 0.004 | 0.027 | ± | 0.038 | 0.033 |  | 0.053 | 0.003 | ± | 0.005 | 0.094 | ± | 0.074 |
| *ido2* |  |  | 0.005 | ± | 0.009 | 0.007 | ± | 0.008 | 0.002 | ± | 0.001 | 0.030 | ± | 0.031^ab^ | 0.008 | ± | 0.010 |  | 0.001 | ± | 0.001 | 0.009 | ± | 0.013 | 0.017 | ± | 0.024 | 0.003 | ± | 0.003^b^ | 0.020 | ± | 0.015 |
| *afmid* |  |  | 0.008 | ± | 0.016 | 0.010 | ± | 0.011 | 0.003 | ± | 0.002 | 0.033 | ± | 0.039 | 0.008 | ± | 0.011 |  | 0.004 | ± | 0.005 | 0.011 | ± | 0.016 | 0.006 | ± | 0.005 | 0.006 | ± | 0.008 | 0.021 | ± | 0.015 |

| Two-way ANOVA | | |  |  |  |  |  |  |  |  |
| --- | --- | --- | --- | --- | --- | --- | --- | --- | --- | --- |
| Parameters | | Time | Diet | Time × Diet |  | Time | | | | |
|  |  |  |  |  |  | 0h | 4h | 24h | 48h | 72h |
| *il1β* |  | <0.001 | ns | ns |  | B | B | B | A | B |
| *il10* |  | <0.001 | ns | 0.003 |  | B | B | B | A | B |
| *il8* |  | <0.001 | ns | 0.022 |  | B | B | B | A | B |
| *tgfβ* |  | <0.001 | ns | 0.009 |  | B | B | B | A | B |
| *sod* |  | <0.001 | ns | 0.029 |  | B | B | B | A | B |
| *cox 2* |  | <0.001 | ns | ns |  | B | B | B | A | B |
| *m2cr* |  | <0.001 | ns | ns |  | B | B | B | A | B |
| *ifn-γ* |  | <0.001 | ns | 0.044 |  | B | B | B | A | B |
| *c3zeta* |  | <0.001 | ns | ns |  | B | B | B | A | B |
| *mcsf1r1* |  | <0.001 | ns | ns |  | B | B | B | A | B |
| *cd8β* |  | 0.014 | ns | ns |  | B | AB | AB | A | AB |
| *hsp70* |  | <0.001 | ns | ns |  | B | B | B | A | B |
| *hsp90* |  | <0.001 | ns | 0.049 |  | B | B | AB | A | AB |
| *mmp9* |  | <0.001 | ns | 0.041 |  | B | B | B | A | B |
| *dicent* |  | <0.001 | ns | ns |  | B | B | B | A | B |
| *gr1* |  | <0.001 | ns | 0.022 |  | B | B | B | A | B |
| *mif* |  | <0.001 | ns | ns |  | B | B | B | A | AB |
| *casp3* |  | 0.011 | ns | ns |  | B | AB | B | A | AB |
| *ido2* |  | <0.001 | ns | 0.006 |  | B | AB | B | A | AB |
| *afmid* |  | <0.001 | ns | ns |  | B | B | B | A | B |

Values are presented as means ± SD (n=6). P-values from two-way ANOVA (p ≤0.05). If interaction was significant, Tukey post hoc test was used to identify differences in the experimental treatments. Different lowercase letters stand for significant differences among dietary treatments for the same time while different symbols stands for significant differences between times for the same diet. Different capital letters indicate differences among times regardless diets and among diets regardless time.

**Table S3.** Forward and reverse primers for real-time PCR.

| Acronym | Gene Bank ID | Eff^1^ | AT^2^ | Product lenght^3^ | Forward primer sequence | Reverse primer sequence |
| --- | --- | --- | --- | --- | --- | --- |
| *ef1α* | AJ866727.1 | 96.45 | 57 | 144 | AACTTCAACGCCCAGGTCAT | CTTCTTGCCAGAACGACGGT |
| *il1β* | AJ311925 | 96.70 | 57 | 105 | AGCGACATGGTGCGATTTCT | CTCCTCTGCTGTGCTGATGT |
| *il10* | [AM268529.1](https://www.ncbi.nlm.nih.gov/entrez/viewer.fcgi?db=nucleotide&id=148472689) | 116.00 | 55 | 164 | ACCCCGTTCGCTTGCCA | CATCTGGTGACATCACTC |
| *il8* | AM490063.1 | 102.87 | 55 | 140 | CGCTGCATCCAAACAGAGAGCAAAC | TCGGGGTCCAGGCAAACCTCTT |
| *tgfβ* | AM421619.1 | 105.56 | 55 | 143 | ACCTACATCTGGAACGCTGA | TGTTGCCTGCCCACATAGTAG |
| *sod* | CX660893.1 | 103.03 | 55 | 71 | GGAGAGTGATTCAGCCCCTG | GGAAACCATGCTCACCAGGA |
| *cox 2* | AJ630649.1 | 81.30 | 61 | 160 | CATTCTTTGCCCAGCACTTCACC | AGCTTGCCATCCTTGAAGAGTC |
| *m2cr* | FR870225.1 | 108.68 | 55 | 676 | GGAACAGGAACCTCCACTCG | ACCACGTGTAGCTGGAACAG |
| *ifn-γ* | FQ310507.3 | 118.38 | 55 | 194 | GTACAGACAGGCGTCCAAAGCATCA | CAAACAGGGCAGCCGTCTCATCAA |
| *c3zeta* | DLAgn_00052540 | 131.01 | 55 | 819 | GCCACCAAAGACACCTACGA | GTGTTGAACGCAGGAGGGTA |
| *mcsf1r1* | DLAgn_00109630 | 125.93 | 55 | 807 | TTGACCGTGGAGAAGGCAAA | AGAATGGACCTCAGCCAGTC |
| *cd8β* | DLAgn_00090370 | 113.81 | 55 | 651 | CGGAACCCAAAAGGCCAAAG | TAGGCTGTAGATGCAGTGCT |
| *hsp70* | AY423555.2 | 134.14 | 55 | 88 | ACAAAGCAGACCCAGACCTTCACCA | TGGTCATAGCACGTTCGCCCTCA |
| *hsp90* | AY395632.1 | 105.63 | 55 | 112 | GCTGACAAGAACGACAAGGCTGTGA | AGATGCGGTTGGAGTGGGTCTGT |
| *mmp9* | FN908863.1 | 98.44 | 57 | 166 | TGTGCCACCACAGACAACTT | TTCCATCTCCACGTCCCTCA |
| *dicent* | AY303949.1 | 89.24 | 55 | 70 | CTCATGGCTGAACCTGGGG | TGGACTTGCCGACGTGAAC |
| *gr1* | AY549305.1 | 103.98 | 55 | 142 | CTTCTACAGCACCAGCACCA | TCTCCTGTTTGACCACACCA |
| *mif* | FN582353 | 123.38 | 60 | 76 | GCTCCCTCCACAGTATTGGCAAGAT | TTGAGCAGTCCACACAGGAGTTTAGAGT |
| *casp3* | DQ345773.1 | 130.10 | 55 | 235 | CTGTGTCGTGACCGCCCT | GATCAAACAGCAAACCCGGC |
| *ido2* | DLAgn_00014730 | 108.20 | 55 | 74 | TGAAGGTGTGAGCAATGAGC | CAAAGCACTGAATGGCTGAA |
| *afmid* | DLAgn_00177950 | 128.26 | 55 | 112 | CGTTTCCACCTGTTTGACCT | CCTAGCCTGCTGAAGGACTG |

1 Efficiency of PCR reactions were calculated from serial dilutions of tissue RT reactions in the validation procedure.

2 Annealing temperature (°C)

3 Amplicon (nt)
